# Exploratory Metabolomic and Metagenomic Profiling of Fecal Samples in Isolated REM Sleep Behavior Disorder

**DOI:** 10.3390/biom16101456

**Published:** 2026-10-06

**Authors:** Fabio Ascari, Luca Baldelli, Daniel Scicchitano, Camilla Pellegrini, Francesco Ravaioli, Sara De Fanti, Erika Esposito, Emanuele Porru, Felice Di Laudo, Raffaele Lodi, Maria Giulia Bacalini, Marco Candela, Federica Provini, Jessica Fiori, Nicolò Interino

**Affiliations:** 1 IRCCS, Istituto delle Scienze Neurologiche di Bologna, Laboratorio di Proteomica Metabolomica e Chimica Bioanalitica, 40139 Bologna, Italy; fabio.ascari@ausl.bologna.it (F.A.); nicolo.interino@ausl.bologna.it (N.I.); 2 Department of Biomedical and Neuromotor Sciences, University of Bologna, 40123 Bologna, Italy; 3 IRCCS, Istituto delle Scienze Neurologiche di Bologna, 40139 Bologna, Italy; 4 Unit of Microbiome Science and Biotechnology, Department of Pharmacy and Biotechnology, University of Bologna, 40126 Bologna, Italy; 5 Occupational Medicine Unit, DIMEC, University of Bologna, 40138 Bologna, Italy; 6 Department of Chemistry “G. Ciamician”, University of Bologna, 40126 Bologna, Italy

**Keywords:** isolated REM sleep behavior disorder, prodromal synucleinopathy, fecal metabolomics, gut–brain axis, short-chain fatty acids, volatilomics, multi-omics integration

## Abstract

Isolated REM sleep behavior disorder (iRBD) is among the most reliable prodromal markers of α-synucleinopathies. Under the body-first (gut-first) model of α-synuclein pathology, in which pathology may originate in the enteric nervous system and ascend to the brain, the gut is a biologically plausible compartment in which early and accessible molecular alterations might be detectable; however, the fecal metabolome remains poorly investigated in this population. In this exploratory, cross-sectional study of 21 iRBD patients and 15 healthy controls, we characterized fecal samples through an integrated multi-omics framework. The fecal metabolome was profiled on two complementary mass spectrometry platforms: HS-SPME GC-EI-high-resolution MS, used for both untargeted volatilomics and targeted quantification of linear and branched short-chain fatty acids, and untargeted LC-MS/MS for the non-volatile fraction. Gut microbial community composition was characterized on the same material by 16S rRNA gene sequencing, and the metabolomic and microbial layers were combined by DIABLO multi-omics integration. Linear short-chain fatty acids were preserved, whereas branched-chain fatty acids were selectively increased in iRBD, indicating a shift toward proteolytic fermentation. Volatile indole was reduced, while p-cresol and 2-octanone were increased, paralleled at the LC-MS/MS level by attenuation of the tryptophan–indole axis, class-level bile acid depletion, and increased acylcarnitines. The microbiome was depleted of *Blautia* and *Faecalibacterium* and enriched in *Oscillospirales*. Integration resolved a coherent host-microbe signature that discriminated iRBD patients from controls within this discovery cohort (84.8% cross-validated accuracy). These cross-sectional findings characterize candidate fecal signatures associated with iRBD; given the exploratory design and the limited sample size, they are hypothesis-generating, and their value for predicting phenoconversion remains to be established in prospective studies.

## 1. Introduction

REM sleep behavior disorder (RBD) is a parasomnia defined by the loss of physiological skeletal muscle atonia during REM sleep, resulting in dream enactment behaviors confirmable by video-polysomnography [1]. Beyond its clinical manifestations, isolated RBD (iRBD) has emerged as one of the most reliable prodromal markers of α-synucleinopathies [2]. Longitudinal cohort studies consistently report that most individuals with a confirmed diagnosis of iRBD eventually convert to Parkinson’s disease (PD), multiple system atrophy (MSA), or dementia with Lewy bodies, with cumulative phenoconversion rates exceeding 70% over follow-up periods of more than a decade [3,4,5]. The interval between iRBD diagnosis and clinical phenoconversion is, however, highly variable, ranging from a few years to more than two decades, and neither the timing nor the specific α-synucleinopathy phenotype to which a given patient will convert can be reliably predicted on the basis of currently available clinical assessments alone [4,6,7,8,9]. This uncertainty represents a major unmet need for individual risk assessment and stratification in iRBD. Intensive research efforts have been directed toward the identification of biomarkers capable of stratifying iRBD patients according to their proximity to phenoconversion and their likely disease trajectory. Candidate biomarkers investigated to date include cerebrospinal fluid α-synuclein seed amplification assays, neuroimaging measures of nigrostriatal dopaminergic integrity, olfactory function, autonomic dysfunction, and motor and cognitive performance [7,10,11,12]. While several of these measures show promise individually, no single biomarker or combination has yet achieved sufficient predictive accuracy for clinical implementation. Furthermore, most available biomarker studies have focused on neurological and systemic readouts, leaving the potential contribution of peripheral biological compartments, particularly the gastrointestinal tract and its microbial ecosystem, relatively underexplored in the iRBD context. The gut–brain axis, defined as the bidirectional network of neural, immune, endocrine, and metabolic communication linking the gastrointestinal tract to the central nervous system, has gained substantial attention as a potential contributor to the pathogenesis and progression of α-synucleinopathies [13,14,15,16]. Gastrointestinal symptoms including constipation are among the earliest non-motor manifestations of PD, frequently antedating motor onset by years, and pathological α-synuclein has been detected in enteric nerve plexuses and appendiceal tissue prior to clinical diagnosis [17,18]. These observations are captured by the body-first (gut-first) model of α-synucleinopathy, which proposes that in a subset of patients misfolded α-synuclein pathology arises in the enteric nervous system and propagates through the vagus nerve to the dorsal brainstem before ascending to the substantia nigra and higher structures [14,16]. Within this framework, the fecal metabolome and its microbial determinants offer an accessible peripheral readout of the intestinal environment in which these early events are hypothesized to occur, providing the rationale for the present study. Gut microbiota dysbiosis has been documented in PD across multiple independent cohorts, characterized at the compositional level by a reduction in short-chain fatty acid (SCFA) producing genera such as *Faecalibacterium*, *Roseburia*, and *Blautia*, and an enrichment of pro-inflammatory taxa including *Enterobacteriaceae* and *Akkermansia* [19,20,21]. Critically, recent cross-sectional studies have shown that gut microbial signatures partially overlapping with those observed in PD are detectable in iRBD, suggesting that dysbiosis may already be present in the prodromal phase and may be useful for future risk stratification of phenoconversion [22,23,24,25].

The gut microbiota exerts its effects on the host largely through the production of bioactive metabolites, whose quantification in fecal samples provides a functional readout of microbial activity that complements compositional sequencing data [26,27]. Among these metabolites, short-chain fatty acids (SCFAs) and branched-chain fatty acids (BCFAs) reflect the balance between saccharolytic and proteolytic fermentation within the gut ecosystem. BCFAs derive from bacterial catabolism of the branched-chain amino acids valine, leucine, and isoleucine, and a relative increase in BCFAs alongside reduced SCFAs has been associated with a shift toward protein-based fermentation in the dysbiotic microbiota reported in PD [25,28,29]. A further pathway of interest is the tryptophan–indole axis, through which commensal bacteria convert dietary tryptophan into indole derivatives such as indole, indole-3-propionic acid, and indole-3-acetic acid, which exert neuroprotective, barrier-stabilizing, and anti-inflammatory effects through aryl hydrocarbon receptor (AhR) and pregnane X receptor (PXR) signaling [30]. In PD and related α-synucleinopathies this protective indole signaling is reported to be attenuated, alongside enrichment of other amino acid-derived microbial catabolites, including the tyrosine-derived compound p-cresol [30,31]. Taken together, these observations indicate that the fecal metabolome encodes biologically interpretable information across the microbiome-host axis of potential relevance to the early pathogenic processes unfolding in iRBD.

Despite growing interest in the gut–brain axis in iRBD, comprehensive multi-platform fecal metabolomic characterizations in this population remain scarce. Most available metabolomic data in iRBD derives from plasma or serum, that capture systemic signals but do not directly reflect local intestinal biochemistry [32]. Moreover, within this setting the volatile fraction of the fecal metabolome has received considerably less attention than its non-volatile counterpart. In PD, VOC-based approaches have so far centered mainly on breath and skin, with fecal volatilomics only recently emerging as a non-invasive readout of gut microbial metabolism [33], and in iRBD the fecal volatilome remains essentially unexplored. This gap is analytically relevant, because several microbially derived volatiles of interest, including indole and short- and branched-chain fatty acids, are poorly retained and detected by reversed-phase LC-MS and are therefore better captured by a dedicated headspace GC-MS volatilomics platform. To our knowledge, no study has combined targeted quantification of linear and branched SCFAs, untargeted GC-MS volatile profiling, and untargeted LC-MS/MS non-volatile profiling on fecal samples from iRBD patients within a single integrated analytical framework.

The present exploratory study aimed to jointly characterize the fecal metabolome and gut microbial community of a cohort of iRBD patients compared with healthy controls, integrating chemical and microbial readouts within a single multi-omics framework applied to fecal samples. Metabolomic profiling was performed using a dual-platform strategy: targeted absolute quantification of linear and branched SCFAs combined with untargeted volatile metabolomics by headspace-solid phase microextraction (HS-SPME) GC-MS, and complementary untargeted LC-MS/MS profiling of the non-volatile fecal metabolome. In parallel, gut microbial community composition was characterized by 16S rRNA gene sequencing on the same fecal material, and the resulting metabolomic and microbial layers were combined through a multi-omics integration approach to identify coordinated host-microbe alterations. Consistent with its exploratory and cross-sectional nature and the limited cohort size, this work is framed as a multi-omics discovery phase: the results are presented as hypothesis-generating findings that characterize the biochemical and microbial landscape of the fecal ecosystem in iRBD and identify candidate features for targeted validation in larger, prospective cohorts. Whether any of these candidates ultimately track α-synuclein-related progression or phenoconversion risk is a question that only longitudinal designs can address.

## 2. Materials and Methods

### 2.1. Study Participants, Clinical Evaluations and Sample Collection

We analysed stool samples from 21 iRBD patients (iRBD) and 15 healthy controls (HC). Participants were selected from our previously described iRBD cohort [34]. Video-polysomnography-confirmed iRBD patients referred to the sleep center of our department were consecutively recruited after exclusion of relevant motor, autonomic, or cognitive impairment at baseline. Absence of systemic or intestinal antibiotic use within 2 months prior to sampling was an inclusion criterion applied to both iRBD patients and healthy controls. Patients underwent standardized clinical and neurophysiological assessments at baseline and annually until phenoconversion or last follow-up. Healthy controls were recruited from the same catchment area as the patients, after excluding motor and cognitive impairment.

Stool samples were self-collected by patients in a screw-cap PP tube and stored at −20 °C. After collection, samples were transported in an insulated container to the research center, where they were aliquoted for subsequent analysis and stored in cryogenic PP tubes at −80 °C until analysis. Because the residual fecal material was insufficient to perform all analytical workflows on every sample, targeted SCFA quantification and GC-MS volatilomics were performed on the full cohort (21 iRBD, 15 HC), whereas LC-MS/MS profiling and 16S rRNA gene sequencing were performed on a subset of 33 participants (19 iRBD, 14 HC); all LC-MS/MS, microbiome and multi-omics integration analyses refer to this subset.

### 2.2. Chemicals and Reagents

Acetic acid (AA), propionic acid (PA), butyric acid (BA), valeric acid (VA), isobutyric acid (iBA), 2-methylbutyric (2mBA), isovaleric acid (iVA), d8-butyric acid (internal standard, IS), perchloric acid, skatole, indole, 2-octanone, p-cresol, C7-C30 saturated alkanes mix, and acid washed glass beads were purchased from Sigma-Aldrich (Milan, Italy). LC-MS grade acetonitrile, LC-MS grade water and LC-MS grade formic acid were purchased by Merck (Darmstadt, Germany). Solid phase microextraction (SPME) DVB/CarbonWR/PDMS fibers, 20 mL headspace (HS) vials and magnetic septum-pierceable caps were purchased from Thermo Fisher Scientific. 2 mL vials PTFE-septum pierceable caps were acquired from Phenomenex (Torrance, CA, USA). For protein removal and final DNA purification DNeasy Blood & Tissue Kit (QIAGEN, Hilden, Germany) was used.

### 2.3. GC-MS Analysis

For GC-MS analysis, an aliquot of approximately 200 mg of feces was weighed into a tube containing acid-washed glass beads to promote mechanical disruption. Perchloric acid (10% aqueous solution) was added to the samples in a constant weight to volume ratio (1:4 *w*/*V*). The suspension was vortexed for 1 min and then centrifuged at 16,000× *g* for 10 min and at 4 °C. A 50 μL aliquot of supernatant was transferred in a 20 mL SPME vial along with 450 μL of pure water and 10 μL of IS. Quality control (QC) samples were prepared by pooling equal aliquots of supernatant of each centrifuged sample. Technical blanks were prepared by substituting the 50 μL aliquot of sample supernatant with water. Samples underwent untargeted GC-MS volatilome characterization by HS-SPME gas chromatography electron impact mass spectrometry (HS-SPME-GC-MS) to investigate naturally occurring volatile organic compounds (VOCs). A TriPlus RSH SMART robotic autosampler was employed to ensure consistent and reproducible pre-analytical VOCs extraction. The SPME fiber used was of the DVB/CarbonWR/PDMS type. Fiber was exposed for 30 min at a temperature of 60 °C under intermittent agitation. VOCs desorption was performed by exposing fiber for 5 min in the GC injection port at a temperature of 250 °C, before splitless injection. The instrument used was a Trace 1610 GC (Thermo Fisher Scientific, Waltham, MA, USA) chromatograph coupled to an Orbitrap Exploris GC-MS mass spectrometer (Thermo Fisher Scientific, Waltham, MA, USA). The GC was equipped with a 30 m × 0.32 mm × 0.25 μm ZBWax standard polar capillary column (Phenomenex, Torrance, CA, USA). Prior to each analysis, an automatic mass calibration was performed during the first minute of the chromatographic run. The temperature program used is here reported: 40 °C held for 5 min, gradient of 20 °C/min to 210 °C, 210 °C held until a total run time of 18.5 min. The carrier gas was grade 5.0 helium (He 99.999%) at a flow rate of 1.0 mL/min. The transfer line between the GC and the mass spectrometer was kept at 240 °C. The ion source operated in EI mode at 70 eV and 300 °C. The C-Trap was maintained at a potential of 0 V. Mass spectra were acquired in full-scan mode over a *m*/*z* range of 40–200 with a resolving power of 60,000 at *m*/*z* 200.

### 2.4. LC-MS/MS Analysis

For each sample, approximately 100 mg of feces was weighed into a tube containing acid-washed glass beads to promote mechanical disruption and maximize extraction efficiency. Cold methanol was added at a constant weight-to-volume ratio of 1:4.5 (*v*/*w*). The suspension was vortexed for 1 min and centrifuged at 16,000× *g* for 10 min at 4 °C. To account for the different starting weights and extraction volumes, an individually recorded aliquot of the methanolic supernatant was taken for each sample and evaporated to dryness in a RapidVac vacuum concentrator at room temperature. Each dried residue was reconstituted in 0.1% formic acid in Milli-Q water at a fixed 4:1 ratio between the volume of evaporated extract and the reconstitution volume, ensuring a constant overall concentration factor across samples regardless of the initial sample weight. The reconstituted extract was sonicated for 5 min, vortexed for 30 s, centrifuged again at 16,000× *g* for 10 min at 4 °C, filtered through a 0.22 µm syringe filter, and transferred to a vial prior to injection. Untargeted profiling of the non-volatile fecal metabolome was performed on an Orbitrap Exploris 240 mass spectrometer coupled to a Vanquish Flex UHPLC system (both Thermo Fisher Scientific, Waltham, MA, USA). Chromatographic separation was achieved on an Accucore aQ C18 Polar Endcapped column (150 × 2.1 mm, 2.6 µm; Thermo Fisher Scientific) maintained at 40 °C. Mobile phase A was water with 0.1% formic acid and mobile phase B was acetonitrile with 0.1% formic acid. The gradient was 0–2 min, 0.5% B; 2–18 min, 0.5–98% B; 18–21 min, 98% B; 21–23 min, 98–0.5% B. The flow rate was 0.350 mL/min and the injection volume was 5 µL. Data were acquired in data-dependent acquisition (full MS/dd-MS2) mode using the AcquireX workflow, with polarity switching to acquire both positive and negative electrospray ionization data within a single run. Full MS spectra were acquired over an *m*/*z* range of 100–1200 at a resolving power of 180,000 at *m*/*z* 200. Pooled QC and solvent blank samples were injected every five samples throughout the analytical sequence for signal monitoring and normalization.

### 2.5. Targeted Data Extraction and Quantification of SCFAs

Targeted quantification of linear and branched SCFAs was performed by slightly modifying a method from the same authors [35]. Briefly, extracted ion chromatograms (XICs) were generated from the same full-scan GC-MS raw data files acquired as described in Section 2.3. Quantification was based on an external calibration approach using an 8-point calibration curve (0.5, 1, 2.5, 5, 10, 20, 40, and 50 µg/mL), with d8-butyric acid 10 µg/mL as internal standard. Calibration curves were constructed with equal weighting, and analyte concentrations were determined by interpolation of analyte-to-IS peak area ratios against the corresponding calibration function. The quantitative method for SCFAs determination was previously fully validated and successfully applied by the same authors [35,36].

### 2.6. Untargeted Volatilomics GC-MS Data Processing

Untargeted processing of the GC-MS full-scan data was performed using Compound Discoverer 3.3 (Thermo Fisher Scientific). Chromatographic deconvolution of the raw data files yielded an initial set of volatile features, which was then progressively reduced through the following sequential filtering steps: background subtraction against solvent blank samples, presence filtering requiring detection in at least 25% of samples within at least one group, removal of silicon-containing features attributed to column bleed or silylation artifacts, and manual exclusion of low-quality or ambiguous chromatographic signals. Metabolite annotation was performed by matching deconvoluted EI spectra against curated spectral libraries and by comparing experimental retention indices, derived from a C7–C30 *n*-alkane series, with tabulated values; the RI was used as evidence orthogonal to the spectral match. Annotation confidence was graded on a five-level scheme (Table 1) adapted from the reporting frameworks of Sumner et al. [37] and Schymanski et al. [38]. The scheme follows the MSI backbone (identified compound, putatively annotated compound, putatively characterized compound class, unknown), with the putative-annotation tier subdivided according to retention index support, and was re-anchored to the evidence accessible under hard ionization: the reproducible EI fragmentation spectrum was treated as the primary diagnostic descriptor, and, because the molecular ion might be weak or absent under EI, class-level inferences were drawn from characteristic fragmentation behavior rather than from the intact precursor. Level 1 required a matching EI spectrum and retention index relative to an authentic reference standard analysed under identical conditions; Level 2, an EI spectral library match supported by a concordant retention index, without a standard; Level 3, an EI spectral library match without retention index confirmation; Level 4, assignment to a compound class from diagnostic EI fragmentation behavior (e.g., an absent molecular ion together with class-characteristic fragment ions), without a specific structural match; and Level 5, features displaying a reproducible, high-quality EI spectrum recurring across samples for which no spectral or structural assignment could be made.

Peak areas were normalized to the extracted ion chromatogram (XIC) area of the deuterium-labeled internal standard (butyric acid-d8) to correct for fiber-related analytical variability across injections. Data were subsequently Pareto-scaled prior to multivariate analysis.

**Table 1:** Annotation confidence definition of the untargeted GC-MS fecal volatilome, graded from Level 1 (identification against an authentic standard) to Level 5 (reproducible but unassigned features); full level definitions are given in Section 2.6.

Confidence Level	Assignment Criterion (Adapted for GC-EI-HRMS)
Level 1	Confirmed identification: EI spectrum and retention index matched to an authentic reference standard (identical conditions).
Level 2	Putative annotation, RI-supported: EI spectral library match with concordant retention index; no authentic standard.
Level 3	Putative annotation: EI spectral library match without retention index confirmation.
Level 4	Putative class assignment: compound class from diagnostic EI fragmentation (e.g., absent molecular ion, class-characteristic fragments); no specific structure.
Level 5	Unassigned feature: reproducible, high-quality EI spectrum recurring across samples; no assignment.

### 2.7. Untargeted LC-MS/MS Data Processing

Raw LC-MS/MS data were processed using Compound Discoverer 3.3 (Thermo Fisher Scientific). Feature detection required a non-zero peak area, the presence of at least one MS2 spectrum, a minimum mzCloud spectral match score, a mass accuracy below 2 ppm, a retention time within the chromatographic window, and a minimum QC peak rating above 8. To reduce analytical noise, features with low variability were further removed by applying an interquartile range (IQR) filtering step, excluding the lowest 30% of features by IQR across the dataset. Signal normalization was performed using the QC-based correction module implemented in Compound Discoverer, using pooled biological QC samples injected periodically throughout the analytical sequence to correct signal drift and batch effects. The resulting feature matrix was subsequently exported to MetaboAnalyst 6.0 for statistical analysis, where data were autoscaled prior to multivariate modeling. Annotation confidence was assigned following the five-level framework of Schymanski et al. [38], with the level-specific assignment criteria applied to the LC-ESI-MS/MS data reported in Table S1.

### 2.8. 16S rRNA Gene Sequencing and Analysis

Microbial genomic DNA was isolated from 250 mg of fecal material using a previously validated protocol [39] with minor modifications. Briefly, samples were subjected to three consecutive cycles of combined chemical, mechanical, and thermal lysis. For chemical disruption, stool samples were suspended in 1 mL of lysis buffer containing 500 mM NaCl, 50 mM Tris-HCl (pH 8.0), 50 mM EDTA, and 4% (*w*/*v*) SDS. Mechanical cell disruption was performed using a FastPrep homogenizer (MP Biomedicals) in the presence of 0.5 g of 0.1 mm zirconia beads and four 3 mm glass beads (BioSpec Products), applying three bead-beating cycles at 5.5 m s^−1^ for 60 s each. Thermal lysis was subsequently achieved by incubating samples at 95 °C for 15 min. Following lysis, particulate debris was removed by centrifugation at 13,000 rpm for 5 min. Nucleic acids were precipitated by adding 260 μL of 10 M ammonium acetate followed by one volume of isopropanol. The resulting pellets were washed with 70% ethanol and resuspended in TE buffer. Residual RNA was eliminated by treatment with DNase-free RNase (2 μL, 10 mg mL^−1^) at 37 °C for 15 min. Protein removal and final DNA purification were carried out using the DNeasy Blood & Tissue Kit according to the manufacturer’s instructions. DNA concentration and purity were determined spectrophotometrically using a NanoDrop ND-1000 instrument (NanoDrop Technologies, Wilmington, DE, USA).

For 16S rRNA gene amplicon library preparation, the V3–V4 region was amplified using the primer pair 341F/785R, modified to include Illumina overhang adapter sequences. Amplicons of the expected size, approximately 460 bp, were purified using Agencourt AMPure XP magnetic beads (Beckman Coulter). Dual-indexed libraries were then generated through a limited-cycle PCR step based on Nextera chemistry and purified again using the same bead-based procedure. Purified libraries were quantified, normalized, and pooled in equimolar amounts. The final pool was denatured with 0.2 N NaOH, diluted to a final concentration of 5 pM, and supplemented with 20% PhiX control. Sequencing was carried out on an Illumina MiSeq instrument using a 2 × 300 bp paired-end configuration, following the manufacturer’s recommendations. Raw paired-end reads were processed using a workflow based on PANDASeq [40] and QIIME 2 [41]. Sequence quality filtering was performed with USEARCH v11 [42], and high-quality reads were denoised into amplicon sequence variants (ASVs) with DADA2 [43], including chimera removal. Taxonomic classification was performed using VSEARCH against the SILVA v138.2 reference database [44], pre-processed with RESCRIPt [45], following the recommendations of the QIIME 2 q2-feature-classifier plugin [46]. Downstream taxonomic profiling and diversity analyses were conducted in QIIME 2 v2023.5. Alpha and beta diversity metrics were computed using the qiime diversity core-metrics-phylogenetic pipeline. Alpha diversity was assessed using Shannon entropy, Faith’s Phylogenetic Diversity (pd) and the number of observed ASVs (observed features), whereas beta diversity was evaluated based on unweighted UniFrac distance, which was subsequently visualized by principal coordinates analysis (PCoA). Graphical representations were generated in R v4.5.3. Differences in alpha diversity were evaluated using the Kruskal–Wallis test, while Linear Discriminant Analysis Effect Size (LEfSe) was used to identify taxa differentially enriched between iRBD patients and healthy controls and to estimate their discriminatory effect size. Statistical significance was set at *p* ≤ 0.05.

### 2.9. Multi-Omics Integration

Multi-omics integration was performed in R using the mixOmics package (6.34.0) through the DIABLO (Data Integration Analysis for Biomarker discovery using Latent cOmponents) framework [47]. Three data blocks were included in the analysis: gut microbiome profiles, LC-MS metabolomics data, and GC-MS volatilomics data. Prior to integration, microbiome data were filtered to remove features with zero variance and low prevalence, retaining taxa detected in at least 5% of samples. To account for the compositional nature of microbiome data, a pseudocount of 10^−6^ was added to all features, relative abundances were calculated, and a centered log-ratio (CLR) transformation was applied. Features showing zero variance after transformation were removed. Metabolomics datasets were independently pre-processed by removing variables with more than 20% missing values. Remaining missing values and zero intensities were imputed using half of the minimum positive value detected for each metabolite. Data were subsequently log10-transformed and variables with zero variance were discarded. Only samples shared across all datasets were retained for downstream analyses. Sample matching among data blocks was verified before model construction.

A supervised DIABLO model was built to discriminate between Healthy controls and iRBD subjects. Relationships among the three data blocks were specified through a design matrix assigning moderate correlations between datasets. Model tuning was performed using the tune.block.splsda function with repeated three-fold cross-validation (20 repetitions) to determine the optimal number of variables (keepX) to retain from each data block. The final model was then fitted using the selected parameters. Model performance was assessed by repeated five-fold cross-validation (50 repetitions) using centroid, Mahalanobis, and maximum distance classification metrics. Classification accuracy was evaluated through overall error rate and balanced error rate estimates. Variables contributing to sample discrimination were identified from the first latent component using the selectVar function, and their relative importance was assessed through component loadings.

To further investigate the multivariate structure of the selected features, a principal component analysis (PCA) was performed on the integrated feature set. Differences between groups were tested by permutational multivariate analysis of variance (PERMANOVA; 999 permutations) using the adonis2 function from the vegan package. Homogeneity of multivariate dispersion was assessed using the betadisper function.

### 2.10. Statistical Analysis and Covariate Handling

Statistical analyses were performed in MetaboAnalyst 6.0 and R v4.5.3.

For all group comparisons, *p*-values were corrected for multiple testing within each dataset using the Benjamini–Hochberg false discovery rate (FDR) procedure; FDR-adjusted *p*-values are hereafter reported as *q*-values.

Age and sex were evaluated as potential confounders. Group differences in age were assessed by *t*-test and differences in sex distribution by Fisher’s exact test. The association between each covariate and each molecular feature was additionally assessed independently of group assignment, using an F-test to evaluate variance homogeneity followed by Student’s or Welch’s *t*-test as appropriate, with Benjamini–Hochberg correction applied within each dataset.

Differential metabolites were then identified using two complementary approaches according to the dataset. For the targeted SCFA data, group differences were assessed by F-test to evaluate variance homogeneity, followed by Student’s or Welch’s *t*-test as appropriate, with correction applied across the seven quantified SCFAs and significance set at *q* < 0.05.

For the untargeted GC-MS and LC-MS/MS datasets, differential features were identified using a Mann–Whitney U test combined with fold-change (FC) evaluation. Consistent with the rank-based, non-parametric nature of the test, fold changes were computed from group medians rather than means. Given the exploratory, hypothesis-generating nature of this study and the limited cohort size, the significance threshold for the untargeted datasets was set a priori at *q* < 0.10 together with |FC| ≥ 2. Features meeting both criteria are reported as candidate discriminant metabolites requiring targeted validation in an independent cohort.

Supervised PLS-DA models were constructed for descriptive purposes only, and model performance was evaluated using *R*^2^, *Q*^2^, and leave-one-out cross-validation accuracy, and its statistical significance was assessed by a permutation test with 1000 label permutations for both classification accuracy and *Q*^2^. Receiver operating characteristic (ROC) curves were generated for candidate discriminatory features, and the corresponding area under the curve (AUC) was computed to assess their individual discriminatory performance. Unless otherwise stated, in all boxplots the central line indicates the median, the box spans the first to third quartiles (interquartile range, IQR), and the whiskers extend to 1.5 times the IQR.

## 3. Results

### 3.1. Cohort Presentation

We recruited 21 iRBD (mean disease duration of 8.61 ± 5.88 years) and 15 HC, Age did not differ significantly between groups (iRBD 71.8 ± 6.5 years vs. HC 69.8 ± 4.4 years; *p* = 0.30). Sex was unevenly distributed between groups, with a higher proportion of males in the iRBD group (14 of 21) than among controls (3 of 15; Fisher’s exact test *p* = 0.008). Over a mean follow-up of 4.88 ± 1.61 years 3 patients phenoconverted to an overt α-synucleinopathy (PD 1 patient, DLB 2 patients).

### 3.2. Covariate Evaluation

No molecular feature was significantly associated with age after FDR correction in either dataset; age was therefore not retained as a covariate in subsequent analyses.

The association between sex and each molecular feature was assessed independently of group assignment: no feature differed significantly between males and females after FDR correction in either the GC-MS or the LC-MS/MS dataset (all *q* > 0.05, Tables S2 and S3).

### 3.3. Targeted Fecal Short Chain Fatty Acids Quantitation

Targeted analysis of fecal SCFAs was first performed to evaluate group-level differences between iRBD subjects and healthy controls (HC, CTRL).

To account for variability in fecal water content across samples, absolute concentrations were normalized by expressing each analyte as a percentage of the total SCFA quantified content within the same sample. Absolute concentrations (µmol/g) are provided in Table S4.

**Figure 1:**
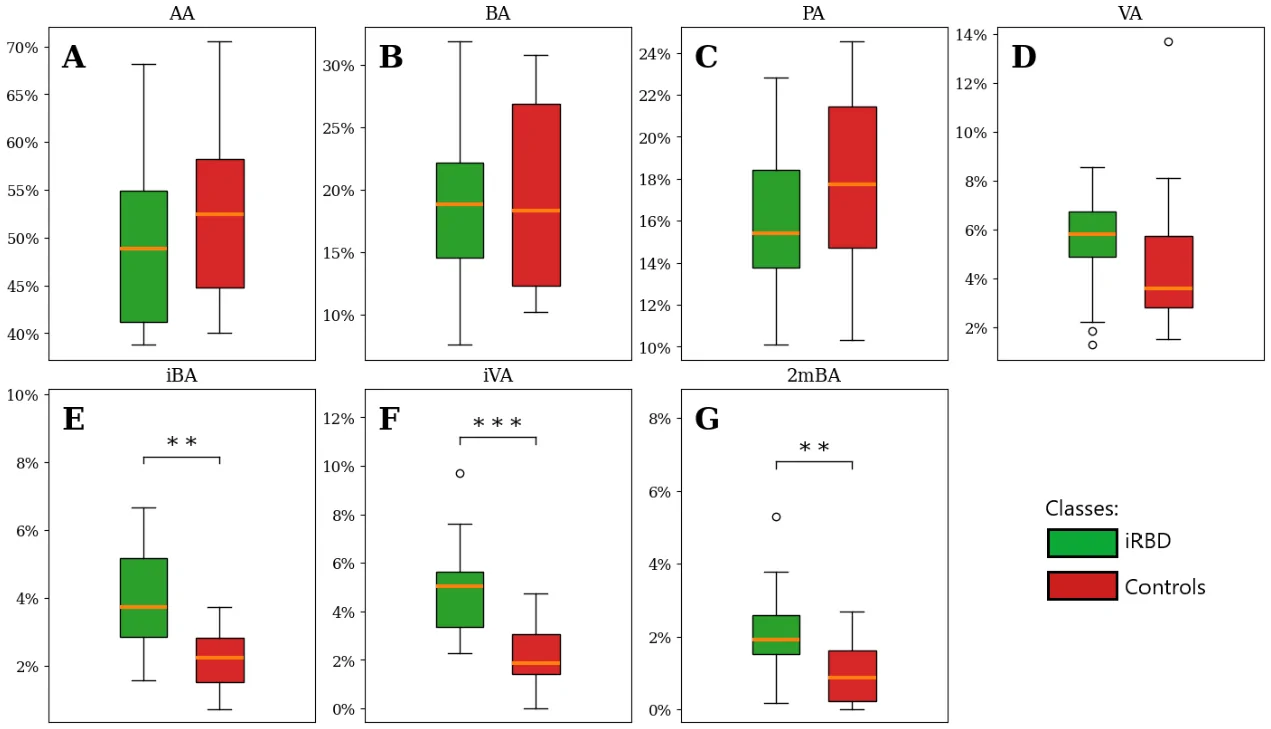
Boxplots showing fecal relative abundances of individual SCFAs in iRBD patients (*n* = 21) and healthy controls (*n* = 15). Linear SCFAs (acetate (**A**), propionate (**C**), butyrate (**B**), and valerate (**D**)) show largely overlapping distributions between groups, whereas branched chain SCFAs (isobutyrate (**E**), isovalerate (**F**), and 2-methylbutyrate (**G**)) are selectively increased in iRBD subjects. Statistical significance is indicated as ** *q* < 0.01 and *** *q* < 0.001 (Student’s or Welch’s *t*-test as appropriate); non-significant comparisons are not marked. Green, iRBD; red, HC.

Univariate statistical analysis revealed no significant differences between iRBD patients and controls for the linear SCFAs: acetic acid, propionic acid, butyric acid, and valeric acid (all *q* > 0.1; Figure 1). In contrast, BCFAs, showed a consistent and statistically significant increase in the iRBD group. Specifically, isobutyric acid, isovaleric acid, and 2-methylbutyric acid relative abundances were significantly higher in iRBD subjects compared with healthy controls (*q* = 1.1 × 10^−3^, 1.8 × 10^−4^, and 5.1 × 10^−3^, respectively).

Mean relative abundances and associated variability for all quantified SCFAs are reported in Table S5. Groupwise distributions are shown in Figure 1, which highlights the selective elevation of branched-chain SCFAs in iRBD, whereas linear SCFA levels largely overlapped between groups.

### 3.4. GC-MS Untargeted Volatilomic Analysis

#### 3.4.1. Exploratory Data Investigation

To further explore fecal metabolic differences beyond targeted SCFAs, an untargeted GC-MS volatilomic approach was applied to the same targeted HS-SPME-GC-MS dataset. Following feature filtering and data pre-processing, exploratory multivariate analysis was performed to assess overall data structure and potential group separation. Across the untargeted HS-SPME GC-EI-Orbitrap dataset, 45 volatile features were retained and graded for annotation confidence on the platform-adapted five-level scheme. 10 features reached Level 1, confirmed against authentic reference standards by matching EI spectrum and retention index; 7 were putatively annotated with retention index support (Level 2) and 6 by spectral library match alone (Level 3). 4 features could be assigned only to a compound class from diagnostic fragmentation behaviour (Level 4), while the largest share, 18 features, consisted of reproducible but unassigned spectra (Level 5). Overall, 23 of 45 features (Levels 1–3) carried a structural annotation, 10 of them confirmed against in-house standards. The full list of GC-MS volatilome features with their assigned confidence levels is provided in Supplementary File 2.

**Figure 2:**
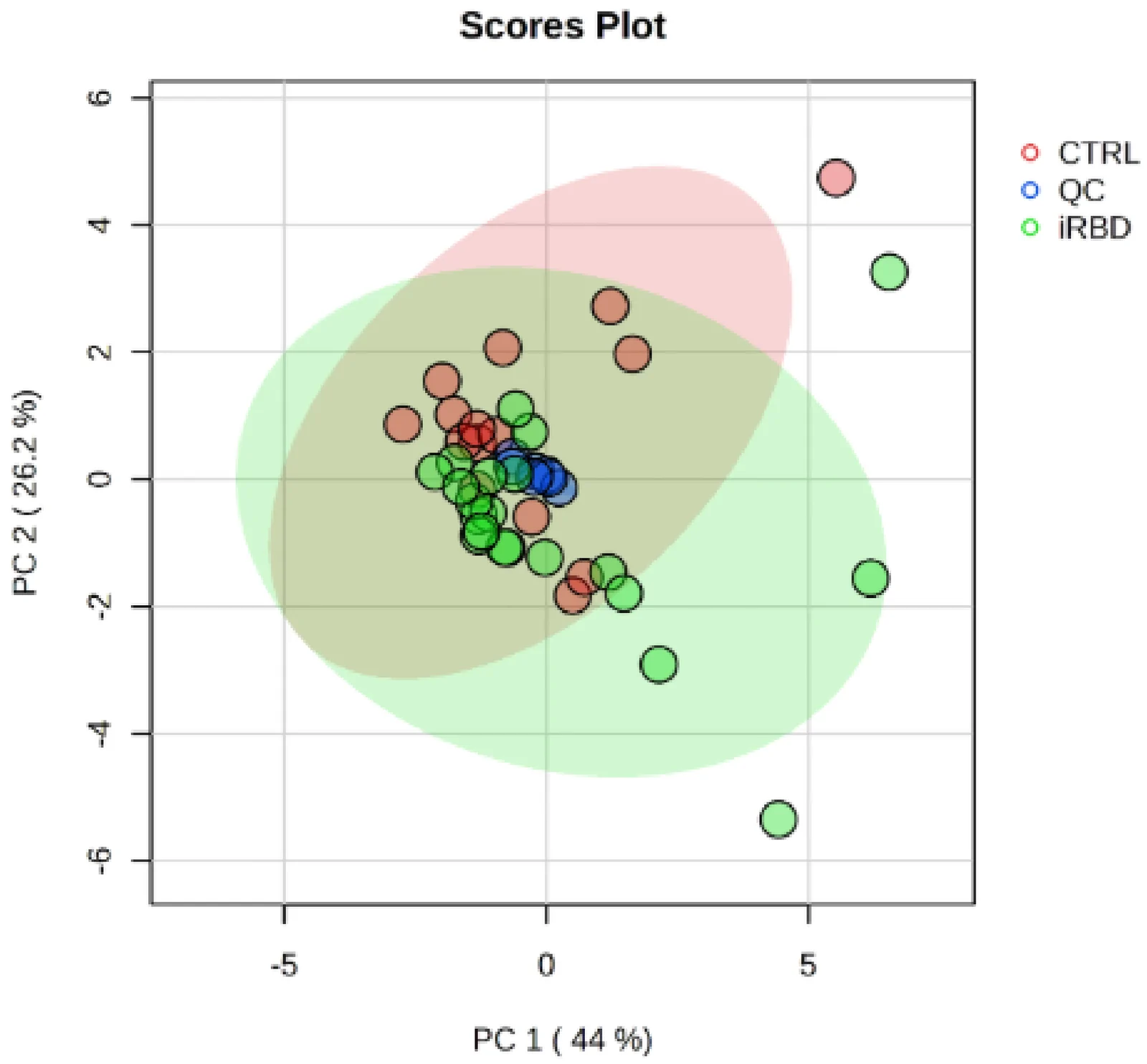
PCA score plot of the untargeted GC–MS volatilomic dataset in iRBD (*n* = 21) and healthy controls (*n* = 15) showing partial group separation between iRBD patients and controls. Green, iRBD; red, HC (labelled CTRL); blue, pooled QC injections; ellipses indicate 95% confidence regions.

PCA on the untargeted results was first used as an unsupervised exploratory tool. The PCA score plot (Figure 2) showed a partial separation between iRBD and control subjects, with a substantial degree of overlap between groups. The first two principal components explained 44.0% (PC1) and 26.2% (PC2) of the total variance, respectively, indicating that a large fraction of volatilomic variability is captured within the low dimensional representation. Within group dispersion was moderate, particularly within the iRBD cohort, consistent with the biological heterogeneity expected in a prodromal neurodegenerative condition. Distribution of feature-wise relative standard deviation (RSD, %) across pooled quality-control injections is reported in Figure S1.

To statistically assess group differences in the multivariate space, a PERMANOVA analysis was performed based on the same data matrix. The analysis revealed a statistically significant separation between iRBD and CTRL groups (*F* = 3.26, *p* = 0.028, 999 permutations) in the PCA (Figure 2). Despite the relatively low R^2^ value, indicating that only around 8.7% of the total variance is explained by group membership, the result supports the presence of a measurable, although modest, metabolic shift associated with the disease condition. This is consistent with multifactorial biological variability typical of fecal metabolomic datasets [48,49]. 

#### 3.4.2. Differential Volatilomic Features

**Figure 3:**
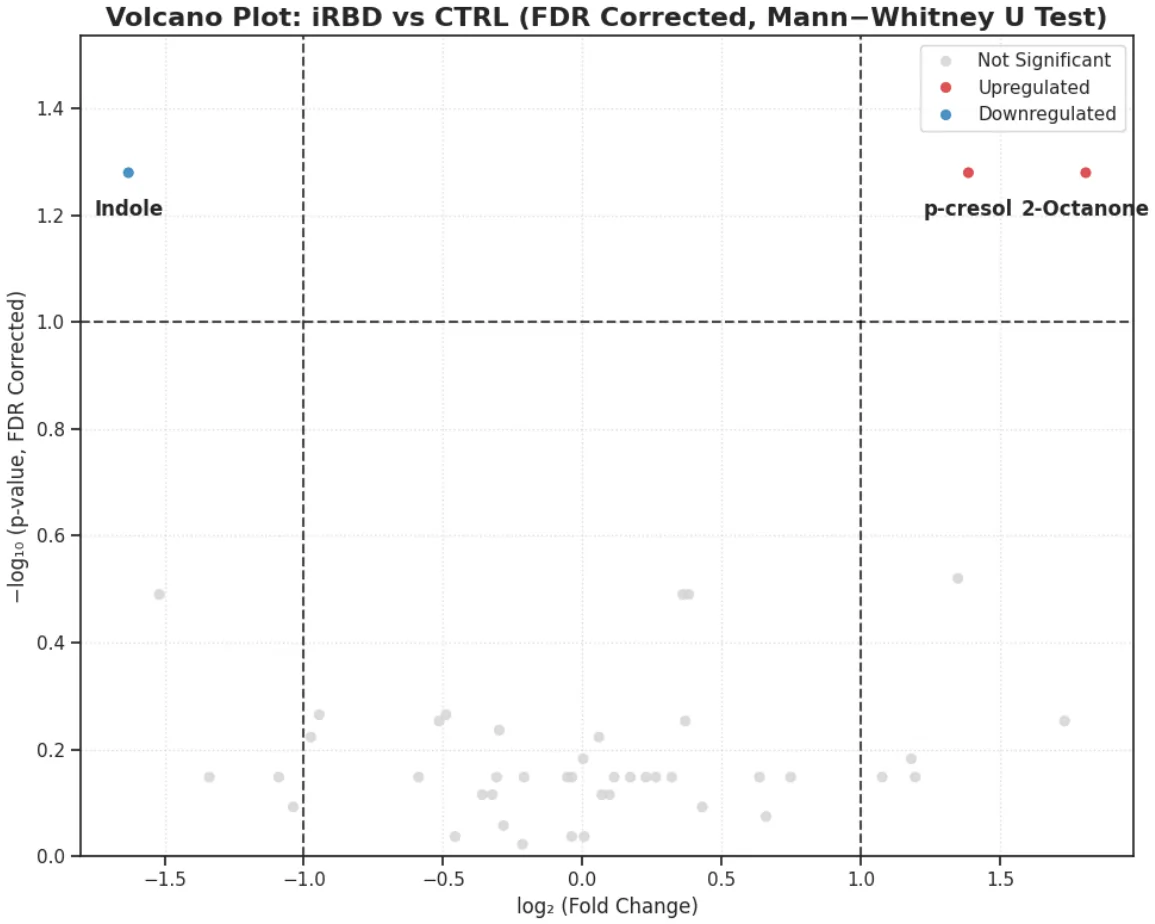
Volcano plot of the untargeted GC–MS volatilome, iRBD (*n* = 21) vs. Controls (*n* = 15). Features meeting the discriminant criteria (*q*-value < 0.10 and |FC| ≥ 2, median-based) are highlighted, with those increased in iRBD shown in red and those decreased in blue. Indole, p-cresol, and 2-octanone were all confirmed against authentic standards (MSI Level 1), excluding mis-annotation for these key metabolites.

To identify individual compounds contributing to group differences, univariate analysis was performed on the GC–MS volatilomic dataset (Section 2.10) and results were visualized through a volcano plot (Figure 3). Three volatiles met the discriminant criteria: 2-octanone was increased in iRBD (FC = 3.5; *p* = 0.0013; *q* = 0.053), p-cresol was increased (FC = 2.6; *p* = 0.0028; *q* = 0.053), and indole was decreased (FC = 0.32; *p* = 0.0035; *q* = 0.053). A marked gap separated these three features from the remainder of the panel, for which the lowest *q*-value was 0.30, indicating a well-defined set of discriminant volatiles rather than a diffuse borderline signal.

Overall, these results identify a small set of candidate discriminant volatiles, supporting the presence of metabolic alterations between iRBD and control subjects, which warrant targeted validation in an independent cohort.

#### 3.4.3. Exploratory Classification Performance

**Figure 4:**
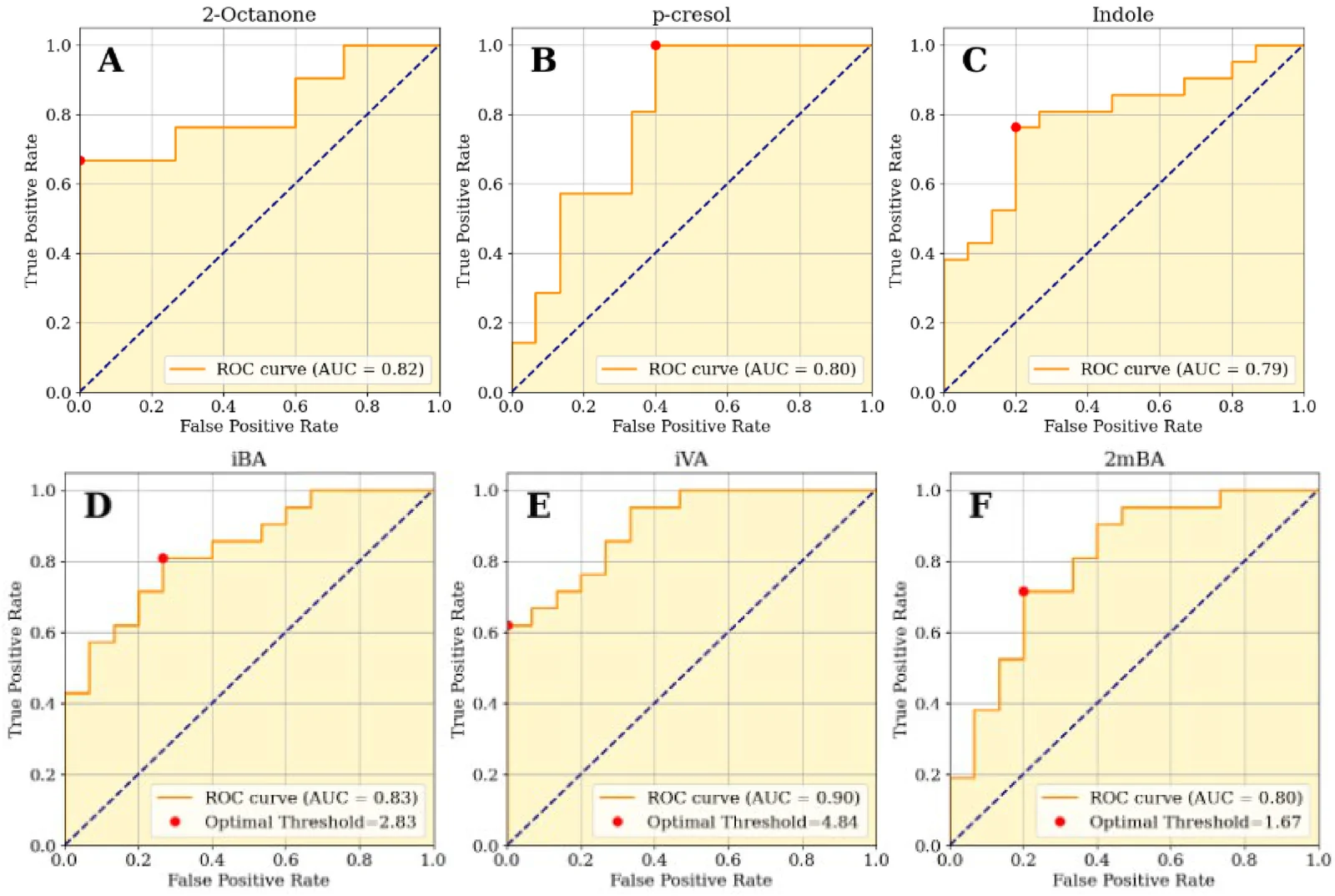
ROC curves for the fecal metabolites that met the applicable significance criteria in the untargeted GC-MS volatilome and targeted SCFA analyses, iRBD (*n* = 21) vs. HC (*n* = 15): (**A**) 2-octanone; (**B**) p-cresol; (**C**) indole; (**D**) isobutyric acid (iBA); (**E**) isovaleric acid (iVA); (**F**) 2-methylbutyric acid (2mBA). The AUC of each curve is reported in the panel legend, and the red dot marks the optimal cut-off; in panels (**D**–**F**) the corresponding threshold (relative abundance, %) is also reported. The dashed diagonal indicates chance-level discrimination (AUC = 0.5). AUC values reflect exploratory, in-sample performance within the discovery cohort and do not represent validated clinical diagnostic accuracy.

ROC curve analysis was performed to evaluate the discriminative potential of the prioritized fecal metabolites, comprising the branched-chain SCFAs (isobutyric, isovaleric, 2-methylbutyric acids) identified by targeted analysis and the features highlighted by the volcano plot of the untargeted GC-MS volatilome: p-cresol, indole and 2-octanone (Figure 4). All features yielded AUC values above 0.8, ranging from 0.8 to 0.9, indicating consistent single-marker discrimination between groups. However, given the limited sample size, these results should be interpreted as purely exploratory and require confirmation in an independent, adequately powered cohort.

### 3.5. LC-MS/MS Untargeted Metabolomic Analysis

#### 3.5.1. Exploratory Data Investigation

Untargeted LC–MS/MS profiling was subsequently employed to characterize non-volatile components of the fecal metabolome and to assess whether broader metabolic alterations were associated with iRBD.

The untargeted LC-ESI-Orbitrap MS/MS dataset comprised 2453 features after IQR filtering, graded following the original Schymanski five-level scheme adaptation (Table S1). As no authentic standards were analysed, Level 1 confirmation was not attainable for this dataset, and no feature was resolved to a formula-only assignment (Level 4). The majority, 1403 features, reached Level 2 through spectral library matching (e.g., mzCloud) without a standard, and 45 features were annotated as tentative candidates (Level 3). The remaining 1005 features corresponded to recurring exact masses of interest that could not be assigned a formula or structure (Level 5). The complete list of LC-MS/MS features with their assigned Schymanski confidence levels is provided in Supplementary File 3.

**Figure 5:**
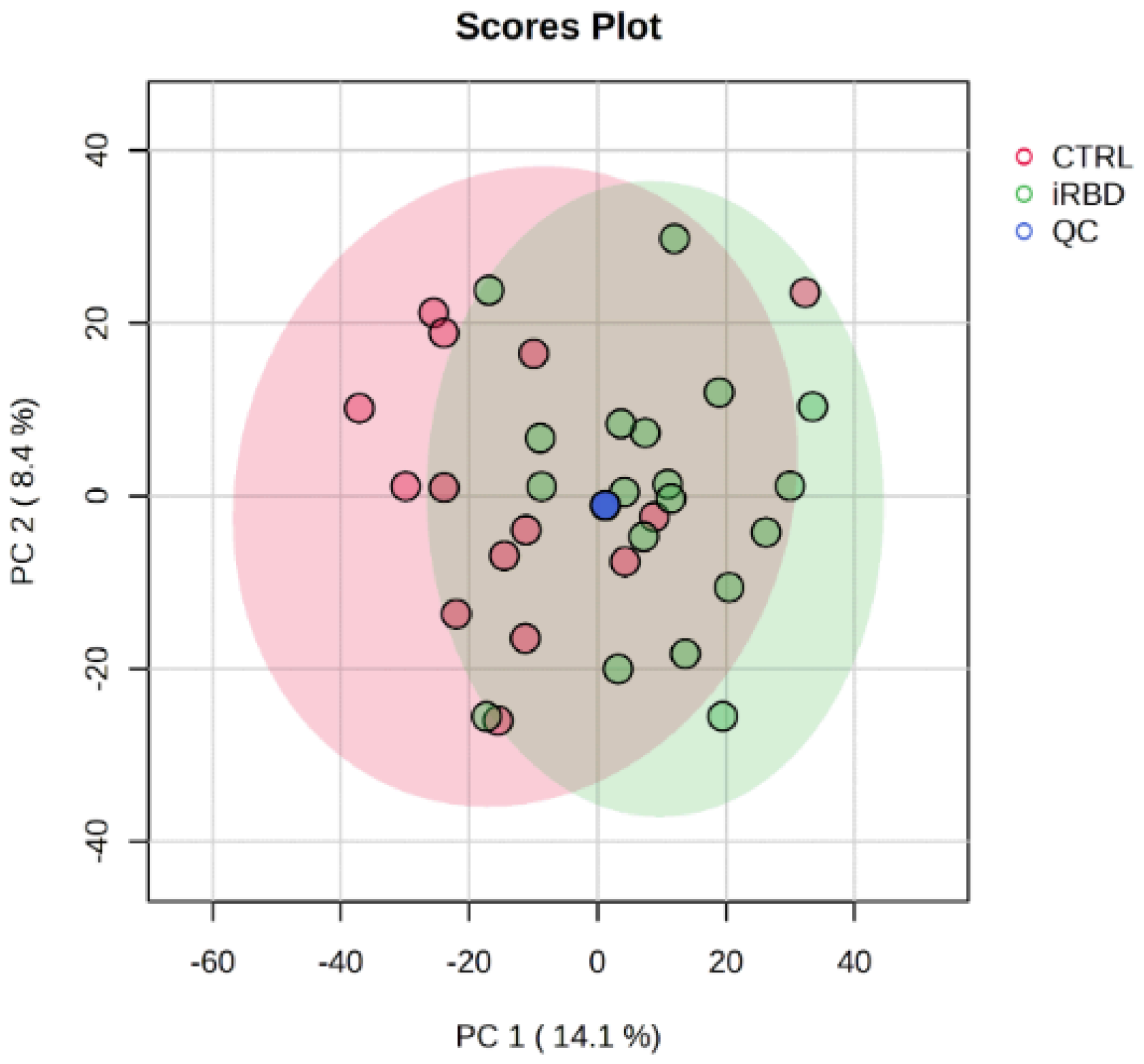
PCA score plot of the untargeted LC–MS/MS dataset showing groupwise separation in iRBD patients (*n* = 19, green) and HC (*n* = 14, red; labelled CTRL) with pooled QC injections (blue); ellipses indicate 95% confidence regions.

PCA of the samples metabolic profile was performed on autoscaled LC–MS/MS data to explore the intrinsic structure of the dataset and to assess analytical stability. Quality control samples showed tight clustering in PCA space, indicating good analytical reproducibility. When considering only biological samples, the PCA score plot revealed a modest but more clearly defined separation between iRBD patients and controls compared with the GC–MS dataset (Figure 5). Distribution of feature-wise relative standard deviation (RSD, %) across pooled quality-control injections is reported in Figure S2.

To statistically validate group-level differences in multivariate space, PER-MANOVA was applied to the LC–MS/MS feature matrix. The analysis demonstrated a significant separation between iRBD and control subjects (*F* = 7.34, *R*^2^ = 0.191, *p* = 0.001; 999 permutations), indicating that approximately 19% of the total variance in the LC–MS/MS metabolome was associated with disease status. 

#### 3.5.2. Differential LC–MS/MS Features

**Figure 6:**
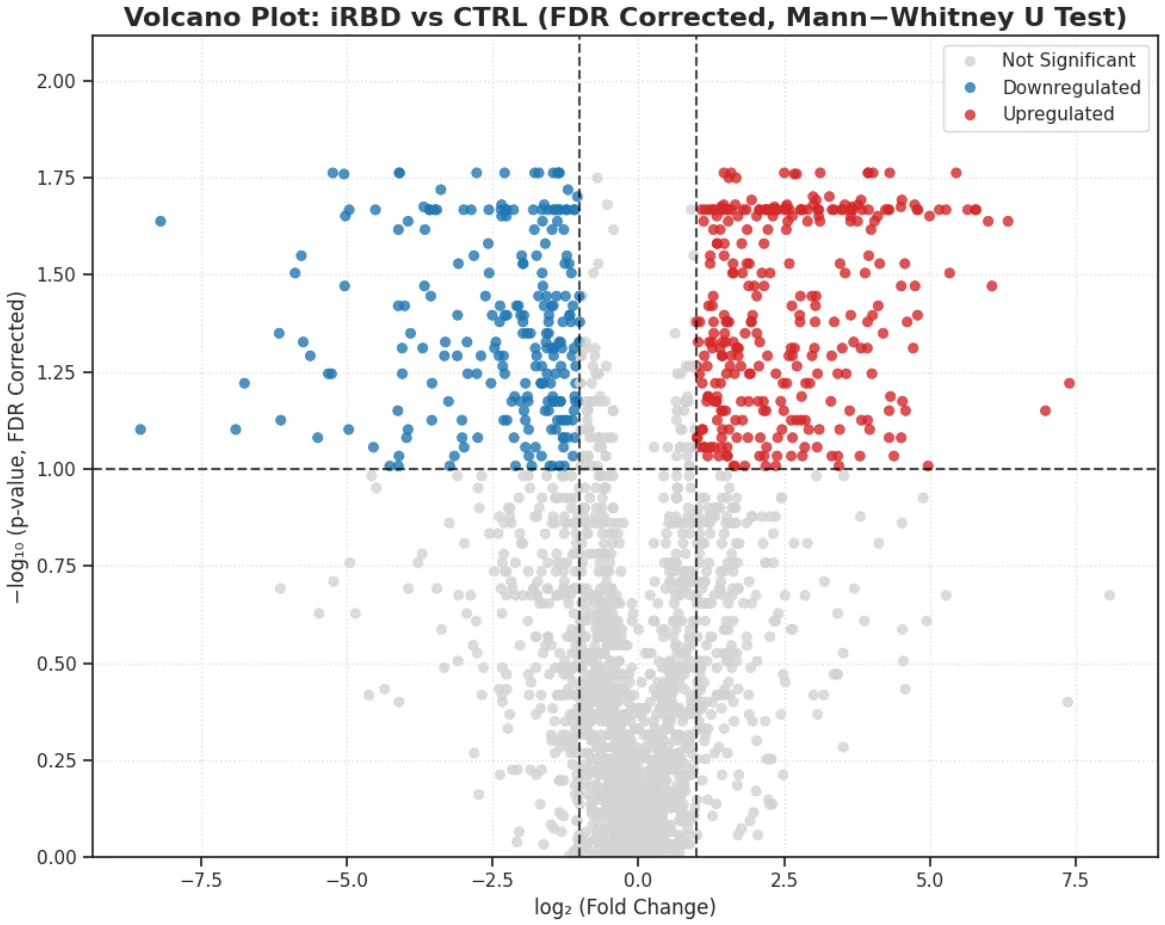
Volcano plot of the untargeted LC-MS/MS dataset comparing iRBD patients (*n* = 19) with controls (*n* = 14). Differential features were identified by Mann–Whitney U test with Benjamini–Hochberg FDR correction; features meeting *q* < 0.10 and |FC| ≥ 2 are highlighted, with those increased in iRBD shown in red and those decreased in blue.

Differential analysis of the untargeted LC-MS/MS dataset was performed as described in Section 2.10, comparing iRBD with CTRL (Figure 6). Applying the discriminant criteria (*q* < 0.10 and |FC| ≥ 2), 539 features were significantly altered in iRBD, 309 increased and 230 decreased; 264 carried a putative annotation, while the remainder corresponded to unannotated *m*/*z* features. Twelve annotated features, corresponding to five compounds assigned to multiple chromatographic features with discordant fold-change directions (likely reflecting isomeric or adduct-level ambiguity), were excluded from biological interpretation, leaving 252 annotated features mapping to 221 unique compounds.

Among annotated features, several biologically coherent classes emerged. The most consistent signal was a uniform reduction in bile acids: all eleven annotated bile acid species were significantly decreased, including chenodeoxycholic acid 3-sulfate (log_2_FC = −4.97), β-muricholic acid (−4.12), taurolithocholic acid 3-sulfate (−3.66), glycochenodeoxycholic acid (−2.82), taurocholic acid (−2.50) and cholic acid (−2.34), with no bile acid feature increased. Tryptophan metabolism was likewise attenuated, with L-tryptophan (log_2_FC = −1.21; *q* = 0.019), its microbial catabolite indole-3-acetic acid (log_2_FC = −1.57; *q* = 0.049), and the tryptophan-containing dipeptide Trp-Pro (log_2_FC = −2.77; *q* = 0.017) all reduced, paralleling the decrease in volatile indole observed by GC-MS. Small di- and tripeptides were predominantly decreased (31 of 39 annotated peptides). Conversely, free carnitine and medium-chain acylcarnitines were consistently increased, including DL-carnitine (log_2_FC = +3.28; *q* = 0.020), O-heptanoylcarnitine (+3.12; *q* = 0.023) and L-hexanoylcarnitine (+4.52; *q* = 0.020), alongside medium-chain dicarboxylic acids (suberic acid, +1.44; azelaic acid, +1.66) and pristanic acid (+6.34; *q* = 0.023). A subset of significant annotations corresponded to xenobiotic and drug-derived metabolites (e.g., phenacetin, imipramine and perindoprilat conjugates, isoetharine); these are consistent with interindividual differences in medication and diet rather than gut microbial metabolism and were not considered further in the biological interpretation.

The 50 differentially regulated LC-MS/MS features with the lowest FDR-corrected *p*-values are reported in Table S6.

#### 3.5.3. Supervised Modeling and Feature Prioritization

**Figure 7:**
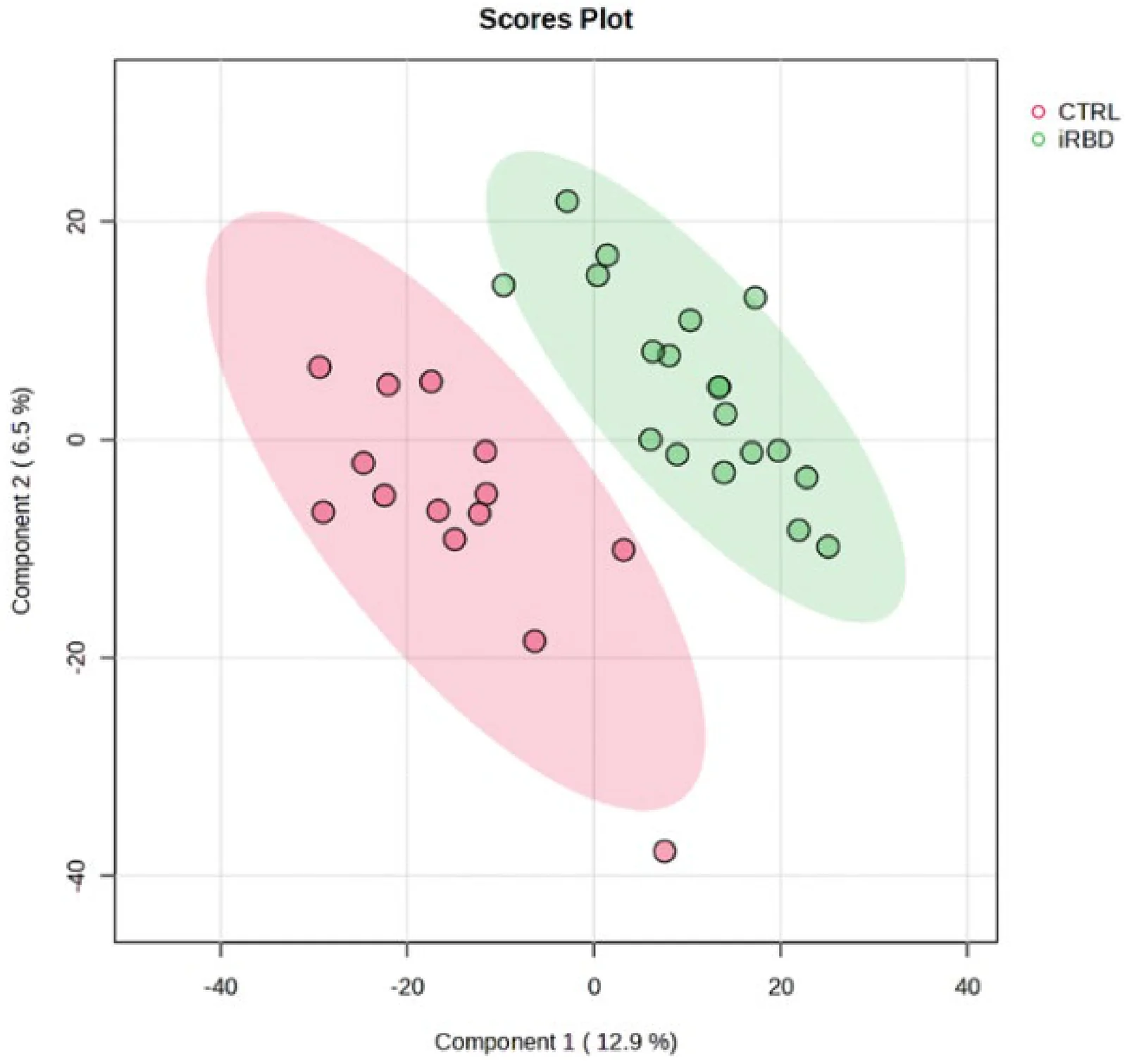
PLS-DA score plot of the untargeted LC-MS/MS dataset showing groupwise separation between iRBD patients (*n* = 19) and controls (*n* = 14). Model validity was confirmed by leave-one-out cross-validation (R^2^ = 0.88, *Q*^2^ = 0.318, accuracy 0.818) and by a 1000-permutation test (*p* = 0.003 for accuracy, *p* = 0.001 for *Q*^2^), supporting that the observed separation is not an artifact of overfitting. Given the limited sample size and the marked difference between *R*^2^ and *Q*^2^ values, the PLSDA model was interpreted as descriptive rather than predictive, and no claim of generalizability to independent cohorts was made. Variable Importance in Projection (VIP) analysis indicated that the observed separation was primarily driven by metabolites such as His–Pro, L-tryptophan, and Trp–Pro, all exhibiting VIP scores greater than 2.0 (Figure S4).

To further explore class separation in the LC–MS/MS dataset, a partial least squares discriminant analysis (PLS-DA, Figure 7) model was constructed as a descriptive tool. The model was built using two latent components and evaluated by leave-one-out cross-validation (LOO-CV). The model showed a high descriptive capacity (*R*^2^ = 0.88) and a moderate predictive ability *(Q*^2^ = 0.318), with an overall classification accuracy of 0.818 under leave-one-out cross-validation. To assess whether this separation could have arisen by chance, class labels were permuted 1000 times: both the classification accuracy and the *Q*^2^ were significantly better than the corresponding permuted null distributions (*p* = 0.003 and *p* = 0.001, respectively), indicating that the model captured genuine group-related structure rather than overfitting (Figure S3).

### 3.6. Gut Microbiota Profiling by 16S rRNA Gene Sequencing

#### Microbial Diversity and Community Structure

**Figure 8:**
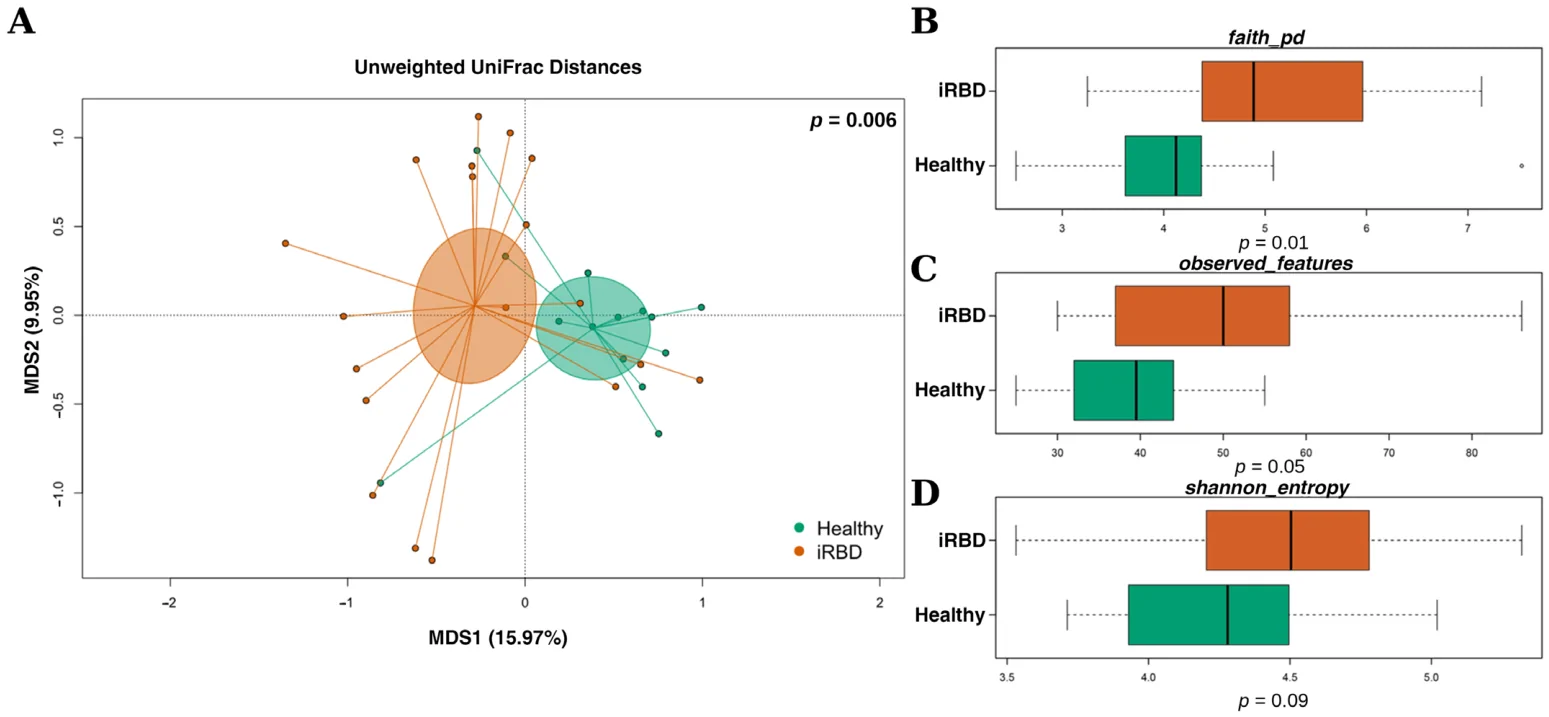
Gut microbial diversity in iRBD patients (*n* = 19) and controls (*n* = 14). (**A**) Beta diversity (unweighted UniFrac PCoA) and alpha diversity metrics (Faith_pd (**B**), observed features (**C**), Shannon (**D**)) are shown for the two groups. Orange, iRBD; green, HC (labelled Healthy).

Beta diversity analysis based on unweighted UniFrac distances of the gut microbiome composition revealed significant differences in gut microbial community structure between individuals with iRBD and healthy controls, Figure 8A. Principal coordinates analysis (PCoA) showed a clear tendency toward group separation, primarily along the first coordinate (MDS1), which explained 15.97% of the total variance. The overall difference in microbial community composition between groups was statistically significant (PERMANOVA, *p* = 0.006). Alpha-diversity analysis (Figure 8B,D) revealed differences in community richness and diversity between groups. In two of the evaluated metrics (faith_pd and observed_features, Figure 8B and Figure 8C respectively), individuals with iRBD exhibited a significantly higher median value than healthy controls (Kruskal–Wallis test, *p* ≤ 0.05), whereas Shannon entropy index did not highlight any significant difference. Overall, the results support the presence of a distinct gut microbial signature associated with iRBD, characterized by alterations in the phylogenetic composition of the intestinal microbiota.

Indeed, LEfSe analysis identified several bacterial genera that significantly discriminated between iRBD patients and healthy controls (Figure S5). The iRBD group was characterized by a significant (Wilcoxon test, *p* < 0.05) enrichment of members of the order *Oscillospirales*, including *UCG-002*, *NK4A214* group, and *UCG-005*, as well as *Christensenellaceae_R-7_group*, *Alistipes*, and the *Eubacterium ventriosum* group, all showing high LDA scores (>3.5). Conversely, healthy subjects exhibited a significant (*p* < 0.05) enrichment of *Blautia, Faecalibacterium*, *Haemophilus*, and *Rothia* genera. Among these, *Blautia* and *Faecalibacterium* displayed the highest discriminative power in favor of the healthy group.

### 3.7. Multi-Omics Integration of Fecal Metabolome and Gut Microbiota

To investigate coordinated alterations across metabolic and microbial layers, an integrative multi-omics analysis was performed using the DIABLO framework implemented in the mixOmics package. Integration included GC–MS volatilomics, LC–MS/MS metabolomics, and 16S rRNA gene sequencing data, restricting the analysis to the 33 participants for whom all datasets were available (19 iRBD, 14 HC).

**Figure 9:**
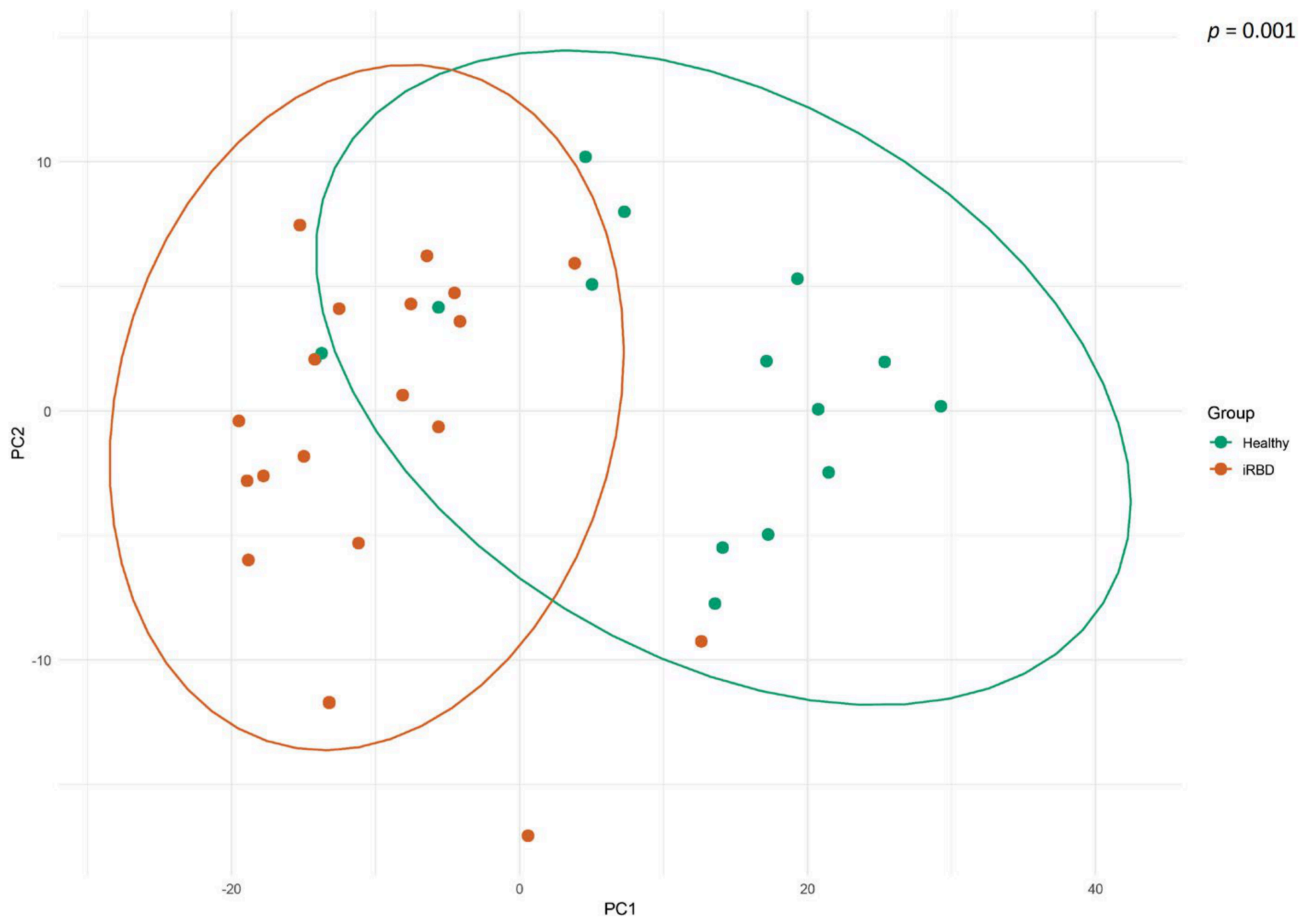
PCA sample score plot generated from the features selected by the DIABLO sparse Partial Least Squares Discriminant Analysis (sPLS-DA) model across the three integrated datasets (genus-level microbiome, LC-MS/MS metabolomics, and GC-MS volatilomics). Each point represents an individual sample and is colored according to the study group (Healthy, green, *n* = 14; iRBD, orange, *n* = 19). 95% confidence ellipses for each group. The separation between Healthy and iRBD groups was significant (PERMANOVA, *p* = 0.001), whereas no significant differences in multivariate dispersion were observed between groups (betadisper ANOVA, *p* = 0.45), indicating that the observed separation was not driven by unequal within-group dispersion. DIABLO predictive performance was assessed using five-fold cross-validation repeated 50 times. Using centroid distance and weighted voting, the model achieved an accuracy of 84.8%, an overall error rate of 0.164, and a balanced error rate of 0.164. Model significance was further confirmed by a 1000-iteration label-permutation test based on cross-validated performance (permutation *p* = 0.001 for both overall error rate and balanced error rate).

Model performance metrics indicated that the integrated DIABLO framework effectively captured disease-associated variation across the three omics layers. The model achieved an overall accuracy of 84.8%, with sensitivity and specificity values of 84.2% and 85.7%, respectively. Moreover, the first latent components derived from the microbiome, LC-metabolomics, and GC-volatilomics datasets exhibited strong concordance (*r* = 0.909, *r* = 0.787, and *r* = 0.834 for pairwise comparisons), indicating that the selected features jointly contributed to a coherent multi-omics signature distinguishing iRBD patients from healthy controls (Figure 9). Feature-selection stability, computed as the frequency with which each variable was retained across the five-fold cross-validation repeated 50 times (250 fitted models), is reported for all selected features in Supplementary File 4.

#### 3.7.1. Global Multi-Omics Structure and Sample Discrimination

The DIABLO model was tuned by cross-validation to identify a limited set of co-varying features from each omics layer that maximized covariance between datasets while preserving group discrimination. The resulting latent component revealed a significant separation between iRBD patients and healthy controls in the integrated multi-omics space, as shown in the PCA (Figure 9; PERMANOVA, *p* = 0.001). Although a partial overlap between groups was observed, the significant PERMANOVA result together with the absence of differences in multivariate dispersion (betadisper ANOVA, *p* = 0.45) indicates that group separation was driven by differences in multivariate composition rather than by unequal within-group variability.

**Figure 10:**
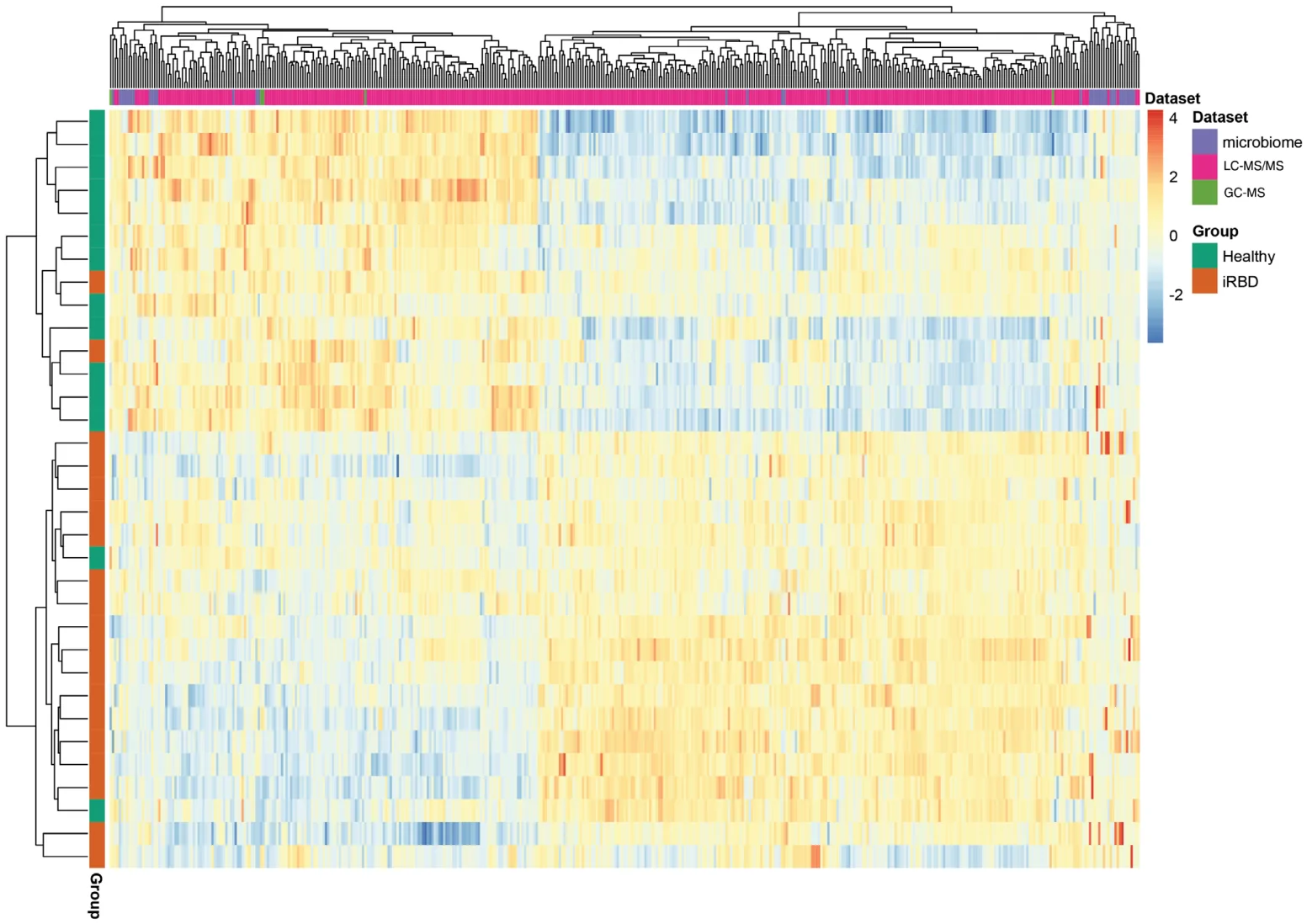
Heatmap of the discriminative biomarkers selected by the DIABLO model across the three integrated datasets (genus-level microbiome, LC-MS/MS metabolomics, and GC-MS metabolomics). Columns represent the selected features, whereas rows correspond to individual samples (*n* = 33; 19 iRBD, 14 HC), annotated according to the study group (Healthy and iRBD) and the originating dataset. Feature abundances were standardized as z-scores prior to visualization, with blue and orange indicating relatively low and high standardized values, respectively. Hierarchical clustering of both samples and features was performed using Euclidean distance and complete linkage, revealing distinct multi-omics signatures associated with the Healthy and iRBD groups. (group: dark green, Healthy; orange, iRBD; dataset: purple, microbiome; magenta, LC-MS/MS; green, GC-MS).

To further investigate the integrated multi-omics signature identified by DIABLO, a clustered image map (CIM) was generated using the features selected on the first latent component (Figure 10). Hierarchical clustering of both samples and variables revealed the presence of distinct multi-omics patterns associated with disease status. Samples tended to segregate according to clinical group, with most healthy controls and iRBD subjects forming separate clusters, although a limited degree of overlap was observed. The heatmap highlighted coordinated variation across microbiome at genera level, LC-metabolomic, and GC-metabolomic features, with several variables displaying opposite abundance patterns between groups. These results support the existence of a coordinated multi-layer molecular signature associated with iRBD and are consistent with the separation observed in the ordination analysis and the significant PERMANOVA results.

#### 3.7.2. Cross-Omics Feature Associations

The cross-block correlation structure among the features retained by the DIABLO model (Figure S6) was dominated by a single coherent module linking the three omics layers. Volatile indole, quantified by GC-MS, acted as the central node: it was inversely associated with a set of LC-MS/MS metabolome features, including the acylcarnitines carnitine (*r* = −0.86), butyrylcarnitine (*r* = −0.83) and hexanoylcarnitine (*r* = −0.83) together with pantothenic acid (*r* = −0.81), and positively associated with the tryptophan-containing dipeptide Trp-Pro (*r* = +0.83). The same LC-MS/MS features were positively associated with two iRBD-enriched microbial genera of the order *Oscillospirales* (*UCG-002* and *UCG-005*; *r* up to +0.74), which were in turn inversely associated with volatile indole (*r* = −0.71 and −0.70) and with Trp-Pro (*r* = −0.72 and −0.71).

Read together with the platform-level findings, the direction of these associations is internally consistent: the *Oscillospirales* genera enriched in iRBD, the reduction in volatile indole, and the increase in short- and medium-chain acylcarnitines co-varied as a single axis, while the tryptophan-containing dipeptide moved in the opposite direction. This convergence reinforces, at the level of coordinated multi-omics behavior, the tryptophan–indole and acyl-metabolism signals identified independently on each platform. The module is anchored by well-annotated, biologically interpretable Level 1 or 2 features, which carry its biological interpretation; the remaining participating features were unannotated (Level 5) and represent additional, still-unidentified candidates for future targeted identification.

To test whether the integrated signature was driven by the sex imbalance, we refitted the DIABLO model after regressing out the effect of sex from each feature, keeping all other parameters unchanged. The sex-adjusted model preserved the same structure and still separated iRBD patients from healthy controls, with a cross-validated accuracy approximately 12 percentage points lower than the corresponding unadjusted run. Importantly, the cross-omics module linking *Oscillospirales* enrichment, reduced volatile indole and increased acylcarnitines was retained in direction, indicating that it is not an artefact of the sex imbalance.

## 4. Discussion

In this exploratory, multi-platform study, individuals with iRBD showed a pattern of metabolic alterations detectable at the prodromal stage of α-synucleinopathies. Data from targeted quantification of linear and branched SCFAs, untargeted GC–MS volatilomics and LC–MS/MS profiling, are collectively consistent with early functional metabolic changes involving microbial amino acid fermentation and downstream metabolite signaling. This interpretation is supported by the preservation of linear SCFAs alongside changes in amino acid–derived metabolites with reported neurobiological relevance.

A consistent finding across targeted analyses was the selective elevation of BCFAs, including isobutyric, isovaleric and 2-methylbutyric acid, in iRBD, in the absence of significant differences in linear SCFAs (acetic, propionic and butyric acid). As BCFAs arise predominantly from microbial fermentation of branched-chain amino acids, their increase is commonly interpreted as a marker of enhanced proteolytic fermentation. The dissociation between preserved linear SCFA and increased BCFAs may indicate that saccharolytic fermentation, and the barrier-supportive functions associated with it, remain largely intact at this stage, with a relative shift toward protein-based microbial metabolism. This pattern differs from that typically reported in PD, where reductions in overall SCFAs availability, particularly butyrate-producing pathways, are common; one possible interpretation is that iRBD reflects an earlier stage of gut metabolic imbalance, although this remains to be tested longitudinally.

Within this amino acid–derived metabolite framework, p-cresol is of particular interest, given its established role as a gut microbiota–derived neurotoxic catabolite of tyrosine [50] and its reported effects on neurobiological pathways relevant to gut–brain communication [51,52]. Experimental work has reported that p-cresol and its sulfate conjugate can affect catecholaminergic systems by inhibiting tyrosine hydroxylase and dopamine-β-hydroxylase activity, thereby reducing catecholamine synthesis, with effects described as more pronounced in brainstem nuclei [53,54]. Whether such mechanisms operate at the exposure levels inferred from fecal profiling remains to be established.

These observations are of potential relevance to iRBD, in which early dysfunction of brainstem catecholaminergic circuits has been documented and linked to REM sleep dysregulation, autonomic impairment, and affective disturbances. It is tempting to speculate that gut-derived p-cresol could act less as a primary neurodegenerative trigger than as a permissive factor exerting effects in already vulnerable brainstem regions. This interpretation is not tested by the present cross-sectional data: p-cresol was measured only in the fecal compartment, its systemic and central exposure were not assessed, and the associations reported here are correlational. The proposed link between fecal p-cresol and brainstem catecholaminergic vulnerability should therefore be regarded as hypothesis-generating and warrants targeted validation, including measurement of circulating and brain-penetrant p-cresol species.

The most pronounced volatile alteration in our cohort was the increase in 2-octanone (FC = 3.5; *q* = 0.053), a finding not previously reported in iRBD. Altered fecal volatile profiles have been increasingly described in PD, where volatilomic signatures have been proposed as non-invasive markers linked to gut microbial dysbiosis [33]. 2-Octanone belongs to the family of C8 fungal volatile organic compounds, which have been experimentally linked to dopaminergic vulnerability: in a Drosophila model, the structurally related semiochemical 1-octen-3-ol induced dopamine neuron degeneration through disruption of dopamine handling, and 2-octanone was included in the same panel of fungal volatile toxicants [55]. The neurotoxic potency demonstrated in that model was, however, highest for 1-octen-3-ol rather than for 2-octanone itself, so this parallel should be regarded as suggestive rather than mechanistic.

At the LC-MS/MS level, disease-associated variation was more pronounced than in the GC-MS volatilome, with group membership accounting for approximately 19% of the variance (PERMANOVA *R*^2^ = 0.19), and differentially regulated metabolites spanning several chemical classes, including peptides, lipid-derived amides, and conjugated bile acids. The most consistent signal was a broad, concordant reduction in bile acid features. Because bile acids comprise numerous positional and stereochemical isomers that share the same molecular formula and yield isobaric signals, and given that annotation was at Level 2 in the absence of authentic standards and without complete chromatographic resolution of these isomers, the individual species underlying each feature could not be unambiguously assigned; a subset of features likely represent redundant annotations of the same compound. The data therefore support a diffuse, class-level depletion of the bile acid pool rather than the confident identification of specific decreased compounds. This class-level reduction is of interest given reports that secondary bile acids participate in the regulation of epithelial integrity, immune tone, and neuroinflammatory signaling, but its functional significance in iRBD remains to be determined. This bile acid contraction, together with the parallel decrease in L-tryptophan and its microbial catabolite indole-3-acetic acid, concordant with the lower volatile indole detected by GC-MS, is compatible with a coordinated attenuation of microbial amino acid and bile acid transformations in iRBD, although the functional consequences cannot be established from cross-sectional fecal data. Free carnitine and medium-chain acylcarnitines were increased in iRBD, whereas butyryl- and succinylcarnitine were annotated to multiple chromatographic features with discordant directions and were therefore not interpreted. Several di- and tripeptides changed bidirectionally and did not define a coherent class trend and are therefore not interpreted further. Finally, several differential features corresponded to drug- and diet-derived xenobiotic metabolites; as medication and dietary intake were not controlled, these are more plausibly attributable to confounding than to disease biology and were excluded from mechanistic interpretation.

At the microbial compositional level, the 16S rRNA data were coherent in direction with the metabolic findings. iRBD subjects were depleted of the saccharolytic, butyrogenic genera *Blautia* and *Faecalibacterium*, which carried the highest discriminatory weight toward the control group, and were enriched in members of the *Oscillospirales*, the *Christensenellaceae*, *Alistipes*, and the *Eubacterium ventriosum* group. The depletion of *Blautia* and *Faecalibacterium* is one of the most reproducible features of the reported PD microbiome and is directionally consistent with the relative reorientation of fermentation toward proteolytic pathways inferred from the rise in BCFAs. Several of the enriched taxa have been associated with amino-acid-oriented and bile-tolerant metabolic niches [56], directionally consistent with the increase in BCFAs; it is worth noting that *Alistipes* is an important marker of proteolytic metabolism. The parallel reduction in fecal bile acids and of tryptophan–indole metabolites is descriptively concordant with this restructuring, although the present design does not allow these associations to be assigned to individual taxa. The higher phylogenetic richness recorded in iRBD (Faith’s_pd and observed features), driven largely by low-abundance, slow-growing taxa, should be interpreted with caution and does not by itself denote a healthier community.

Integrating these layers, the supervised DIABLO model resolved a coordinated host-microbe signature separating iRBD from controls with a cross-validated classification accuracy of 84.8% (sensitivity 84.2%, specificity 85.7%). The first latent components of the microbiome, LC–MS/MS, and GC–MS blocks were associated (r = 0.91, 0.79, and 0.83 for the pairwise comparisons), consistent with compositional and metabolic changes that co-vary along a shared axis rather than occurring independently. The corresponding taxon-to-metabolite associations are detailed in Section 3.7.2. Given the exploratory design, the limited cohort, and the feature-selection step intrinsic to DIABLO, these integrated patterns are presented as internally consistent and hypothesis-generating rather than as independently validated, and the reported classification metrics should not be interpreted as an estimate of diagnostic performance in an independent population. Because sex was strongly collinear with clinical group, this adjustment (described in Section 3.7.2) also removes part of the disease-associated signal rather than isolating an independent sex effect. We therefore retained the unadjusted model (84.8% cross-validated accuracy) as the primary analysis and present the sex-adjusted model as a conservative sensitivity analysis; validation in a larger, sex-balanced cohort will be required to disentangle the contributions of sex and disease status.

This work configures an exploratory, hypothesis-generating study rather than a confirmatory or predictive one, and three features of its design define the scope of its conclusions. First, the approach was deliberately data-driven: three untargeted platforms were profiled without a pre-specified molecular hypothesis, and candidate discriminant features were identified through statistical screening and multivariate feature selection, with the biological interpretation developed around the features that emerged. This strategy is well suited to charting a compartment not previously examined in iRBD, most notably the fecal volatilome, but it carries an intrinsic risk of chance associations and overfitting given the number of features relative to the cohort size; accordingly, the multivariate models are treated as descriptive, the cross-platform convergence is presented as internally coherent rather than independently validated, and every discriminant feature is framed as a candidate requiring targeted confirmation. Second, the study evaluates clinically defined iRBD at a single cross-sectional time point and does not track phenoconversion: with only three patients converting over the available follow-up, these data cannot establish whether any signature predicts progression to an overt α-synucleinopathy, a question that only prospective longitudinal designs can resolve. Third, dietary intake was not assessed, and because diet directly shapes the fecal metabolome, including short- and branched-chain fatty acid production and tryptophan-derived metabolites, unmeasured dietary differences may have contributed to the observed alterations; the presence of diet- and drug-derived xenobiotic features among the discriminant metabolites, excluded from mechanistic interpretation, is consistent with this. These considerations frame the present findings as a discovery-phase map of the iRBD fecal ecosystem and as a source of specific, testable hypotheses for the next, appropriately powered and longitudinal phase.

A few further limitations should be acknowledged. An exploratory FDR threshold of *q* < 0.10 was adopted for the untargeted datasets given the limited sample size, so the discriminant metabolites reported here should be regarded as candidate biomarkers requiring targeted validation in an independent cohort. The control group included a lower proportion of male subjects than the iRBD group, and this imbalance should be considered when interpreting the integrated signature. Finally, direct measurement of circulating or brain-penetrant p-cresol was beyond the scope of the present work. Notwithstanding these constraints, the internal consistency of the metabolomic signal and its alignment with established mechanisms of gut–brain interaction support the relevance of the findings.

Because gut microbial composition can be influenced by medication, we reviewed the pharmacological history of the iRBD cohort to assess its potential contribution to the observed differences. Since antibiotic use in the 2 months before sampling was an exclusion criterion in both groups, antibiotic-driven distortion of the fecal microbiome is an unlikely explanation for the group differences. The most frequently recorded medications were RBD-directed symptomatic therapy (clonazepam and/or melatonin, 57%) and cardiovascular agents (57%), followed by antithrombotics (48%), whereas proton-pump inhibitors (19%) were comparatively infrequent. Isolated exposures with recognized effects on gut microbial ecology, such as metformin and a tyrosine-kinase inhibitor, were each limited to a single patient and therefore could not account for group-level signals. Healthy controls were not receiving any continuous or chronic pharmacological therapy, although occasional as-needed use of over-the-counter drugs cannot be excluded.

## 5. Conclusions

In this exploratory study, iRBD was associated with a fecal metabolomic profile characterized by enhanced proteolytic fermentation, attenuation of the tryptophan–indole axis, and early disturbance of amino acid and bile acid related metabolism, occurring in the absence of marked depletion of linear short-chain fatty acids. Three volatile metabolites emerged as candidate discriminants, with p-cresol and 2-octanone increased and indole decreased, while multi-block integration of the metabolomic and microbial layers resolved a coherent host-microbe signature separating iRBD from controls.

Within this framework, p-cresol is of particular interest as a gut microbiota-derived catabolite with reported effects on catecholaminergic systems, and therefore represents a biologically plausible, though yet untested, effector at the prodromal stage. Its relevance to brainstem vulnerability in iRBD remains hypothesis-generating and requires measurement of circulating and brain-penetrant species.

These results position fecal metabolomics as a sensitive window into early gut–brain metabolic interactions and identify candidate pathways and metabolites for targeted quantitative validation in independent, sex-balanced cohorts, and for longitudinal assessment of their value as biomarkers or mechanistic contributors in prodromal α-synucleinopathies.

## Data Availability

The datasets generated during the current study are available from the corresponding author on reasonable request.

## Supplementary Materials

The following supporting information can be downloaded at https://www.mdpi.com/article/10.3390/biom16101456/s1. Supplementary File 1: Table S1: Annotation-confidence level assignment criteria of the untargeted LC-MS/MS dataset (following Schymanski five-level scheme [38]); Table S2: Pearson *r* correlation between GC-MS feature amounts and sex and relative *p*-values and FDR adjusted *p*-values; Table S3: 50 highest Pearson *r* correlation between LC-MS/MS feature amounts and sex and relative *p*-values and FDR adjusted *p*-values; Table S4: Fecal short chain fatty acid absolute concentrations reported for each sample; Table S5: Fecal short chain fatty acid relative abundances (% of total SCFA content per fecal sample) in iRBD patients and controls; Table S6: 50 highest Mann−Whitney FDR-corrected *p*-values and fold changes on group medians for differentially regulated LC–MS/MS features; Figure S1: Distribution of feature-wise relative standard deviation across pooled quality-control injections for the untargeted GC-MS volatilome platform; Figure S2: Distribution of feature-wise relative standard deviation across pooled quality-control injections for the untargeted LC-MS/MS platform; Figure S3: PLS-DA cross validation results on up to five components number for LC-MS/MS dataset; Figure S4: Best 30 VIP-scores from PLS-DA built on 2 components; Figure S5: LEfSe analysis of 16S rRNA genus-level data showing the bacterial genera that significantly discriminated iRBD patients from healthy controls, with their linear discriminant analysis (LDA) effect-size scores and the group in which each taxon was enriched; Figure S6: Circos plot of the multiblock sparse Partial Least Squares Discriminant Analysis (sPLS-DA) generated using the mixOmics package. Supplementary File 2: GC-MS volatilome features and annotation-confidence levels. Levels follow the platform-adapted five-level EI scheme defined in Table 1 of the main text. Supplementary File 3: LC-MS/MS features and annotation-confidence levels. Levels follow the five-level scheme of Schymanski et al. (2014) [38] defined in Table S1. Supplementary File 4: Feature-selection stability of the DIABLO-selected features.
