# Supplementary material for: Exploratory Metabolomic and Metagenomic Profiling of Fecal Samples in Isolated REM Sleep Behavior Disorder
Source: Biomolecules. 2026 Oct 6;16(10):1456. doi: 10.3390/biom16101456 (PMC13702349; doi:10.3390/biom16101456)
Supplement: Supplementary file 1 [file biomolecules-16-01456-s001.zip › Supplementary File 1.pdf]

Exploratory Metabolomic and Metagenomic Profiling of Fecal Samples in Isolated REM Sleep Behavior Disorder

Fabio Ascari [1], Luca Baldelli [2,3], Daniel Scicchitano [4], Camilla Pellegrini [3], Francesco Ravaioli [3], Sara De Fanti [3], Erika Esposito [1], Emanuele Porru [5], Felice Di Laudo [2], Raffaele Lodi [2,3], Maria Giulia Bacalini [2,3], Marco Candela [4], Federica Provini [2,3], Jessica Fiori [1,6,*] and Nicolò Interino [1]

[1] IRCCS, Istituto delle Scienze Neurologiche di Bologna, Laboratorio di Proteomica Metabolomica e Chimica Bioanalitica, Bologna, Italy;
[2] Department of Biomedical and Neuromotor Sciences, University of Bologna, Bologna, Italy;
[3] IRCCS, Istituto delle Scienze Neurologiche di Bologna, Bologna, Italy;
[4] Unit of Microbiome Science and Biotechnology, Department of Pharmacy and Biotechnology, University of Bologna, Bologna, Italy;
[5] Occupational Medicine Unit, DIMEC, University of Bologna, Bologna, Italy;
[6] Department of Chemistry "G. Ciamician", University of Bologna, Bologna, Italy; jessica.fiori@unibo.it;

* Correspondence: jessica.fiori@unibo.it;

Table S1. Annotation-confidence level assignment criteria of the untargeted LC-MS/MS dataset (following Schymanski five-level scheme [38]).

Confidence level	Assignment criterion (LC-ESI-MS/MS)
Level 1	Identity confirmed against an authentic reference standard analysed under identical conditions (MS^2 spectrum + retention time).
Level 2	Probable structure: MS^2 spectral-library/database match (e.g., mzCloud), without an authentic standard.
Level 3	Tentative candidate: single candidate structure proposed but not confirmed (database/in-silico match), or annotation by analogy to a known compound (mass difference, 'Similar to').
Level 4	Unequivocal molecular formula assigned from accurate mass and isotope pattern, without a defined structure.
Level 5	Exact mass of interest (and retention time) only; no formula or structure assigned.

Table S2. Pearson r correlation between GC-MS feature amounts and sex and relative p-values and FDR adjusted p-values. Level, annotation-confidence level of each feature on the platform-adapted five-level EI scheme (Table 1 of the main text; full list in Supplementary File 2).

Feature	Correlation	p-value	FDR p-value	Level
Ethyl butyrate	-0.34	0.043	0.934	2
@13.95	-0.31	0.067	0.934	5
2-Octanone	0.26	0.124	0.934	1
p-cresol	0.26	0.126	0.934	1
Ethyl valerate	-0.24	0.155	0.934	2
Isovaleric acid	0.23	0.174	0.934	1
@12.94	0.22	0.198	0.934	5
2-Heptanone	-0.22	0.201	0.934	2
Indole	-0.21	0.223	0.934	1
Phenylethanol	0.20	0.236	0.934	3
Maleimide, 2-ethyl, 3-methyl-	0.20	0.244	0.934	3
@17.808	0.20	0.249	0.934	5
C6H12O2	-0.19	0.277	0.939	4
2-methylbutyric acid	0.18	0.301	0.939	1
@14.111	0.17	0.321	0.939	5
Butanoic acid	-0.16	0.352	0.939	1
C11H22O	0.13	0.436	0.939	4
Dimethyl trisulfide	-0.13	0.441	0.939	2
@17.998	0.13	0.448	0.939	5
@14.853	-0.13	0.449	0.939	5
@15.073	-0.12	0.469	0.939	5
@14.263	0.12	0.484	0.939	5
Heptanoic acid	0.12	0.497	0.939	2
Isobutyric acid	0.12	0.501	0.939	1
Naphthalene, dihydro, trimethyl-	-0.10	0.570	0.947	3
Indole, 3-methyl-	0.09	0.583	0.947	3
Furfural, 5-methyl-	0.09	0.613	0.947	3
@15.188	0.08	0.643	0.947	5
@10.378	0.07	0.696	0.947	5
Benzene, tetramethyl-	0.07	0.699	0.947	3
C9H18O2	-0.06	0.721	0.947	4
@15.596	-0.06	0.726	0.947	5
Propanoic acid	-0.06	0.732	0.947	1
C15H30O	0.06	0.741	0.947	4
@5.116	0.05	0.763	0.947	5
@10.603	-0.05	0.766	0.947	5
@8.264	-0.04	0.799	0.947	5

@10.817	0.04	0.816	0.947	5
Hexanoic acid	-0.03	0.848	0.947	2
@12.261	-0.02	0.888	0.947	5
Limonene	0.02	0.897	0.947	2
@12.206	0.02	0.903	0.947	5
Valeric acid	0.02	0.911	0.947	1
Acetic acid	-0.02	0.926	0.947	1
@14.402	-0.01	0.970	0.970	5

Table S3. 50 highest Pearson r correlation between LC-MS/MS feature amounts and sex and relative p-values and FDR adjusted p-values. Level, annotation-confidence level of each feature on the five-level scheme of Schymanski et al. (Table S.1; full list in Supplementary File 3).

Feature	Correlation	p-value	FDR p-value	Level
Centralite	-0.62	0.000	0.264	2
Gly-Tyr	-0.57	0.000	0.613	2
@11.398-	0.52	0.002	0.618	5
trans-2-Aminomethyl-1-cyclohexanol	-0.51	0.003	0.618	2
@8.044-	0.50	0.003	0.618	5
His-Pro	-0.50	0.003	0.618	2
@16.357+	0.49	0.003	0.618	5
@8.75-	0.49	0.004	0.618	5
N-(Carboxymethyl)-N-(2-((carboxymethyl)amino)ethyl)glycine	-0.49	0.004	0.618	2
Icaridin	-0.48	0.004	0.618	2
@5.296+/-	-0.48	0.005	0.618	5
Pro-Leu	-0.47	0.005	0.618	2
2-Amino-3-hydroxyoctadecyl beta-D-glucopyranoside	0.47	0.006	0.618	2
1b.3a.12a-Trihydroxy-5b-cholanoic acid.1	0.46	0.007	0.618	2
VASICINONE	-0.45	0.008	0.618	2
@13.759+/-	0.45	0.008	0.618	5
@1.63-	-0.45	0.008	0.618	5
N-Acetyl-L-alanine	-0.45	0.008	0.618	2
@1.274+	-0.45	0.009	0.618	5
L-Alanyl-L-proline	-0.45	0.009	0.618	2
Uridine	-0.45	0.009	0.618	2
5-Acetylamino-6-amino-3-methyluracil	0.44	0.010	0.618	2
6-Hydroxy-3.4-dihydro-2(1H)-quinolinone.1	0.44	0.011	0.618	2
@1.215+/-	-0.44	0.011	0.618	5
@9.765-	0.44	0.011	0.618	5
@6.14+	-0.44	0.011	0.618	5
@5.288+	-0.43	0.011	0.618	5
(2Z)-2-(5-hydroxy-7-methyl-3-methylidene-2-oxo-3a.4.5.7.8.8a-hexahydrocyclohepta[b]furan-6-ylidene)acetic acid	-0.43	0.012	0.618	2
Pro-Ser-Ile	-0.43	0.012	0.618	2
@5.899+/-	-0.43	0.012	0.618	5
Inosine.1	-0.43	0.012	0.618	2
Ser-Val	-0.43	0.013	0.618	2
Hypoxanthine	-0.43	0.014	0.618	2
@1.215+	-0.42	0.014	0.618	5

@5.803-	0.42	0.015	0.618	5
Asp-Ala-Ile	-0.42	0.015	0.618	2
Hypoxanthine.1	-0.42	0.015	0.618	2
4-morpholinobenzoic acid	0.42	0.016	0.618	2
Tazettine.1	0.42	0.016	0.618	2
@1.1-	0.42	0.016	0.618	5
2.3-dihydroxyoctanoic acid	0.42	0.016	0.618	2
@1.026+	0.42	0.016	0.618	5
Glycyl-L-leucine	-0.42	0.016	0.618	2
5-Acetylamino-6-amino-3-methyluracil.1	0.41	0.017	0.618	2
@9.114+/-	0.41	0.017	0.618	5
@9.185-	0.41	0.018	0.618	5
5-Acetamido-4-oxohexanoic acid	0.41	0.018	0.618	2
Trp-Pro	-0.41	0.018	0.618	2
@1.264+	-0.41	0.018	0.618	5
Thr-Ile	-0.41	0.018	0.618	2

Table S4. Fecal short chain fatty acid absolute concentrations reported for each sample. All seven SCFAs were identified at confidence Level 1 (EI spectrum and retention index matched to authentic reference standards; Table 1 of the main text).

		SCFA concentration in μmol/g of fecal sample						
**Sample**	**Class**	**AA**	**PA**	**iBA**	**BA**	**iVA**	**VA**	**2mBA**
F11	iRBD	24.3	5.3	2.1	3.6	1.6	0.7	1.0
F12	iRBD	28.4	6.2	1.2	3.4	1.1	0.9	0.5
F13	iRBD	29.9	16.4	4.6	14.5	4.2	4.4	1.4
F2	iRBD	50.8	11.7	5.0	15.8	4.8	4.7	2.5
F14	iRBD	38.3	15.4	2.5	7.1	2.3	0.9	1.0
F15	iRBD	22.4	11.2	2.7	16.0	1.9	3.0	0.1
F22	iRBD	16.2	6.6	1.2	6.7	1.8	2.1	0.9
F23	iRBD	12.4	4.2	1.2	4.0	1.3	0.9	0.5
F24	iRBD	19.3	4.0	1.5	9.2	2.3	2.0	1.3
F25	iRBD	19.4	8.3	0.9	6.4	1.4	2.3	0.7
F26	iRBD	13.8	4.2	0.8	4.6	1.3	1.8	0.5
F27	iRBD	6.0	2.1	0.3	2.6	0.7	0.7	0.2
F28	iRBD	31.7	14.4	4.2	19.0	4.0	5.4	1.5
F29	iRBD	35.0	12.0	1.4	27.4	2.0	7.4	1.0
F30	iRBD	18.7	5.6	1.4	9.6	2.0	3.1	1.0
F31	iRBD	15.6	4.1	2.7	8.9	3.9	3.0	2.1
F32	iRBD	35.3	8.6	2.0	5.2	1.9	1.6	0.8
F33	iRBD	13.4	3.1	0.4	4.7	0.6	1.9	0.2
F34	iRBD	35.7	12.7	3.4	19.3	5.5	7.1	3.1
F35	iRBD	13.2	3.2	0.8	1.7	1.6	1.2	0.6
F36	iRBD	10.9	4.4	1.2	3.5	1.8	1.2	0.9
F7	CTRL	71.6	16.1	1.4	20.8	0.7	2.9	0.1
F8	CTRL	35.5	14.0	1.6	7.2	1.2	2.1	0.2
F9	CTRL	41.2	17.2	2.9	12.1	2.2	1.9	1.1
F10	CTRL	50.5	17.9	1.7	11.1	1.3	2.6	0.6
F21	CTRL	26.3	5.0	0.7	15.1	0.9	1.0	0.0
F1	CTRL	20.3	10.7	1.5	4.9	2.2	6.5	1.3
F37	CTRL	20.3	5.4	0.8	4.0	1.1	1.7	0.6
F38	CTRL	18.8	9.3	1.0	5.4	1.3	1.5	0.7
F39	CTRL	28.5	13.9	2.4	17.8	2.9	4.1	1.7
F42	CTRL	22.0	10.3	0.4	15.0	0.0	1.5	0.0
F3	CTRL	37.8	10.7	1.2	26.0	1.2	6.8	0.7
F43	CTRL	13.6	2.9	0.3	2.0	0.2	0.3	0.0
F44	CTRL	30.1	10.0	1.5	19.9	1.7	4.6	1.1
F45	CTRL	37.0	12.1	1.1	15.2	1.2	2.5	0.7
F46	CTRL	21.3	7.8	1.6	9.5	1.0	2.5	0.4

Table S5. Fecal short chain fatty acid relative abundances (% of total SCFA content per fecal sample) in iRBD patients and controls. Data are reported as mean ± standard deviation. Between group comparisons were performed using two tailed t tests following assessment of variance homogeneity by F test. Level, annotation-confidence level (Table 1 of the main text); all seven SCFAs were identified at Level 1 against authentic reference standards.

SCFA	IRBD (mean % ± SD)	CTRL (mean %± SD)	F test p value	T test p value	Level
Acetate (AA)	49.61 ± 8.80	52.19 ± 8.56	0.94	0.39	1
Propionate (PA)	15.97 ± 3.54	18.03 ± 4.22	0.46	0.12	1
Isobutyrate (iBA)	3.91 ± 1.42	2.23 ± 0.92	0.10	3.1×10^{-4}	1
Butyrate (BA)	18.01 ± 6.54	19.59 ± 7.93	0.42	0.52	1
Isovalerate (iVA)	4.89 ± 1.82	2.22 ± 1.28	0.17	2.6×10^{-5}	1
Valerate (VA)	5.42 ± 2.08	4.69 ± 3.10	0.10	0.40	1
2-Methylbutyrate (2mBA)	2.19 ± 1.13	1.04 ± 0.88	0.34	2.2×10^{-3}	1

Table S6. 50 highest Mann-Whitney FDR-corrected p-values and fold changes on group medians for differentially regulated LC–MS/MS features. Level, annotation-confidence level of each feature on the five-level scheme of Schymanski et al. (Table S.1; full list in Supplementary File 3).

Feature	Log_2 (FC)	FDR p-value	Level
@1.215+	-1.7	0.017	5
@10.036+/-	3.9	0.017	5
@10.375-	-4.1	0.017	5
@9.226+/-	4.0	0.017	5
@8.192+	3.1	0.017	5
Sweroside	-4.1	0.017	2
Ala-Ala-Ala.2	2.5	0.017	2
Glycochenodeoxycholic acid.1	-2.3	0.017	2
@4.457+	4.3	0.017	5
Ala-Ala-Ala.3	1.6	0.017	2
@8.55-	-5.2	0.017	5
@5.876+/-	-1.8	0.017	5
2.3-dihydroxyoctanoic acid	1.5	0.017	2
Tyr-Pro	-1.4	0.017	2
Trp-Pro	-2.8	0.017	2
@5.233+/-	-1.4	0.017	5
@6.255+	3.9	0.017	5
Glu-Glu-Ile	-1.5	0.017	2
His-Pro	-1.4	0.017	2
@6.289+	5.5	0.017	5
@2.852+	2.7	0.017	5
Butyrylcarnitine	2.7	0.017	2
@12.415-	-5.0	0.017	5
@5.537	1.7	0.018	5
@8.307+/-	1.6	0.018	5
L-Tryptophan	-1.2	0.019	2
3-Ketosucrose.1	-3.4	0.019	2
Thr-Pro	-1.0	0.020	2
DL-Carnitine.2	3.3	0.020	2
@6.745+	3.0	0.020	5
@8.741+	1.9	0.020	5
S-(3-oxo-3-carboxy-n-propyl)cysteine	3.1	0.020	2

Feature	Log_2 (FC)	FDR p-value	Level
Ala-Hyp-Gly.2	5.3	0.022	2
(Z)-6.9.10-trihydroxyoctadec-7-enoic acid	-3.6	0.022	2
3-Methylxanthine.2	2.4	0.022	2
5-methoxy N.N-diethyl Hexylone	3.9	0.022	2
VASICINONE	-1.1	0.022	2
@3.993+	5.8	0.022	5
@8.043	2.3	0.022	5
@8.19+	4.2	0.022	5
@3.949+/-	4.8	0.022	5
2-Methylsuccinic acid	-2.4	0.022	2
@8.211+/-	2.1	0.022	5
Calpeptin	2.8	0.022	2
@4.408+	2.8	0.022	5
@1.227+	-2.3	0.022	5
@11.101+	3.1	0.022	5
Pantothenic acid	2.9	0.022	2
@8.107+/-	3.4	0.022	5
@7.553-	2.1	0.022	5
(E)-8.9.10-trihydroxyoctadec-6-enoic acid	-3.6	0.022	2
@5.057+	2.3	0.022	5
@7.708+/-	1.5	0.022	5
@4.107+	3.3	0.022	5
@7.765-	-4.5	0.022	5
@8.021-	2.5	0.022	5
L-2-succinylamino-6-oxoheptanedioic acid	-1.3	0.022	2
Neplanocin A.1	1.4	0.022	2
6.8-dihydroxy-8a-methyl-5-[(E)-prop-1-enyl]-5.6.7.8-tetrahydroindolizin-3-one	2.2	0.022	2
3.4-Pyrrolidinediol. 2-(p-methoxybenzyl)-. 3-acetate. (2S.3R.4R)-.1	2.5	0.022	2
@1.63-	-1.8	0.022	5
carbapenem MM22383	3.7	0.022	2
2-Hydroxyphenylalanine.4	1.3	0.022	2
Methylimidazoleacetic acid	1.6	0.022	2

2-Aminooctanedioic acid.4	3.8	0.020	2
L-Hexanoylcarnitine	4.5	0.020	2
@6.498+	3.5	0.021	5
@5.423+	-1.6	0.021	5
[Similar to: Pro-Ala-Ser; deltaMass: 0.0002 Da]	1.5	0.021	3
@8.852+/-	4.7	0.021	5
JOVAHKVKEDHFJU-ATNNCRSISA-N	3.0	0.021	3
@10.393-	2.6	0.021	5
@9.335+	3.8	0.021	5
Azelaic acid.2	1.7	0.021	2
Glycosmisic Acid	-2.3	0.021	2
@10.738-	-3.7	0.021	5
@9.547+	2.5	0.021	5
@8.912+	3.7	0.021	5
@7.247+	2.3	0.021	5
Asn-Ala-Leu	4.5	0.021	2
Ala-Hyp-Gly.1	1.4	0.021	2
@8.673+	3.1	0.022	5
Decanohydroxamic acid	3.5	0.022	2
@5.728+	2.4	0.022	5
N-Acetylvaline	1.3	0.022	2
4-Piperidinol. 1-hydroxy-2.2.6.6-tetramethyl-.1	1.8	0.022	2
(3E.5S.6S.7S.9R.11E.13E.15R.16R)-16-Ethyl-15-(hydroxymethyl)-5.7.9-trimethyl-2.10-dioxooxacyclohexadeca-3.11.13-trien-6-yl 3.4.6-trideoxy-3-(dimethylamino)-beta-D-xylo-hexopyranoside	-2.9	0.022	2
Myxalamid A	-3.5	0.022	2
Glu-Glu-Ile.1	-1.1	0.022	2
@6.135+	2.3	0.022	5
10-Hydroxy-2-oxabicyclo[6.2.2]dodeca-1(10).8.11-trien-3-one.2	1.2	0.022	2
diethyl oxalpropionate.1	1.4	0.022	2
ITEVFNFZADKMQH-YZHZHMMSSA-N.3	-2.6	0.022	2
@6.549+	2.3	0.022	5
@11.186+/-	-5.0	0.022	5
Dihydrocaffeic acid.1	-1.6	0.022	2
@7.037+/-	-1.7	0.022	5
Tazettine.1	1.9	0.022	2
RFDAIACWWDREDC-ICXMQNBNSA-N	-3.0	0.022	3
@6.844+	5.8	0.022	5

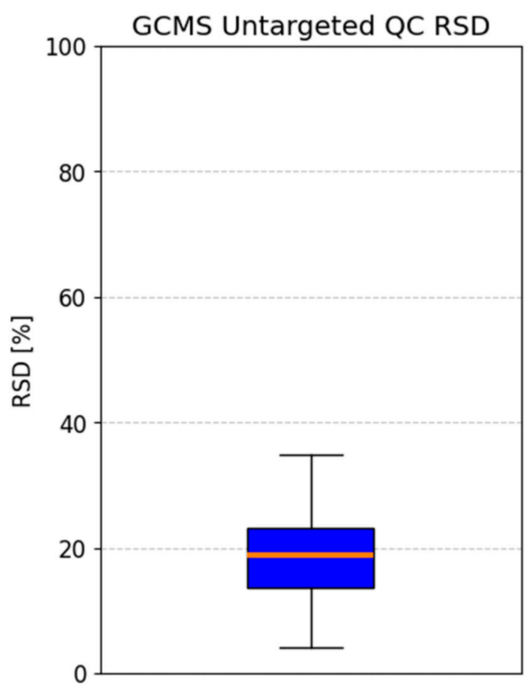

Figure S1. Distribution of feature-wise relative standard deviation across pooled quality-control injections for the untargeted GC-MS volatilome platform.

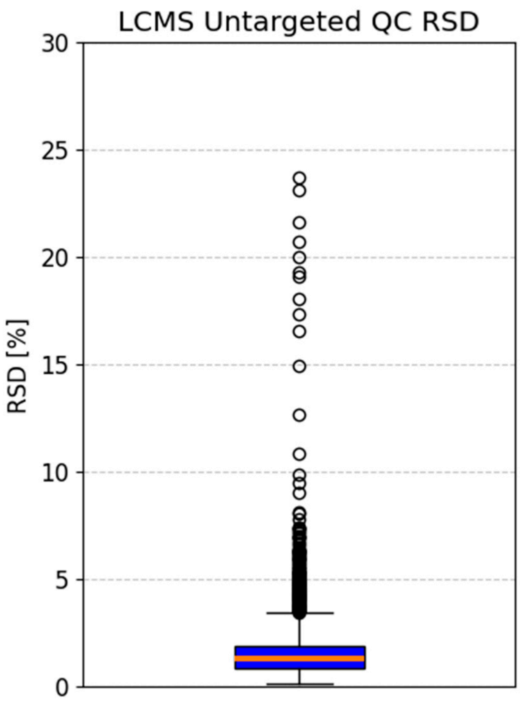

Figure S2. Distribution of feature-wise relative standard deviation across pooled quality-control injections for the untargeted LC-MS/MS platform.

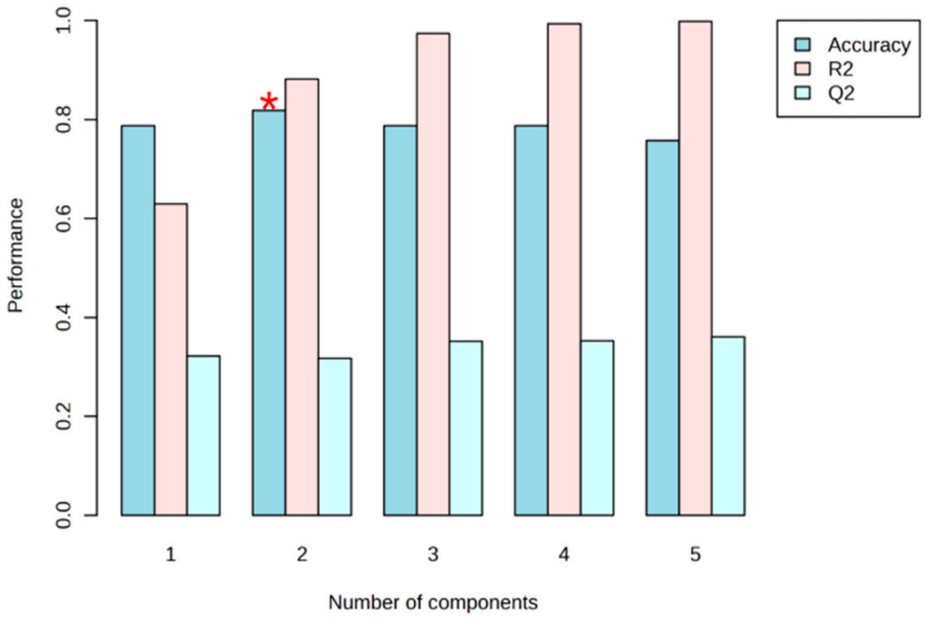

Figure S3. PLS-DA cross validation results on up to five components number for LC-MS/MS dataset

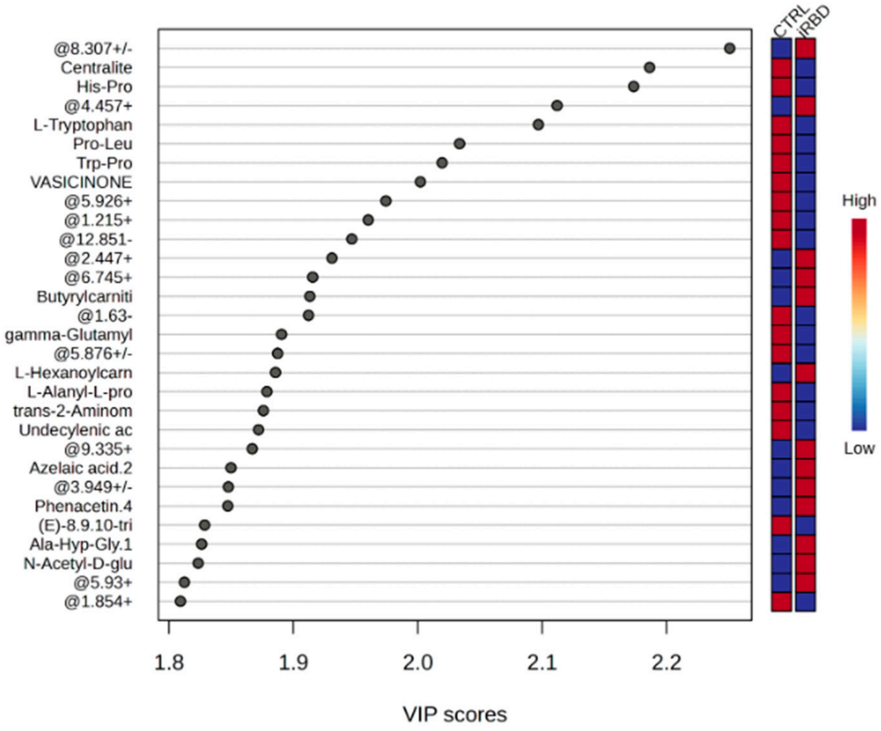

Figure S4. Best 30 VIP-scores from PLS-DA built on 2 components.

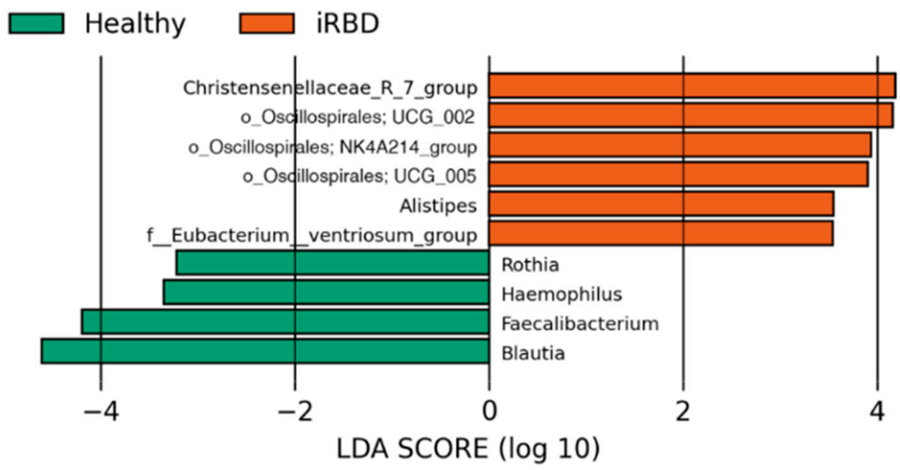

Figure S5. LEfSe analysis of 16S rRNA genus-level data showing the bacterial genera that significantly discriminated iRBD patients from healthy controls, with their linear discriminant analysis (LDA) effect-size scores and the group in which each taxon was enriched.

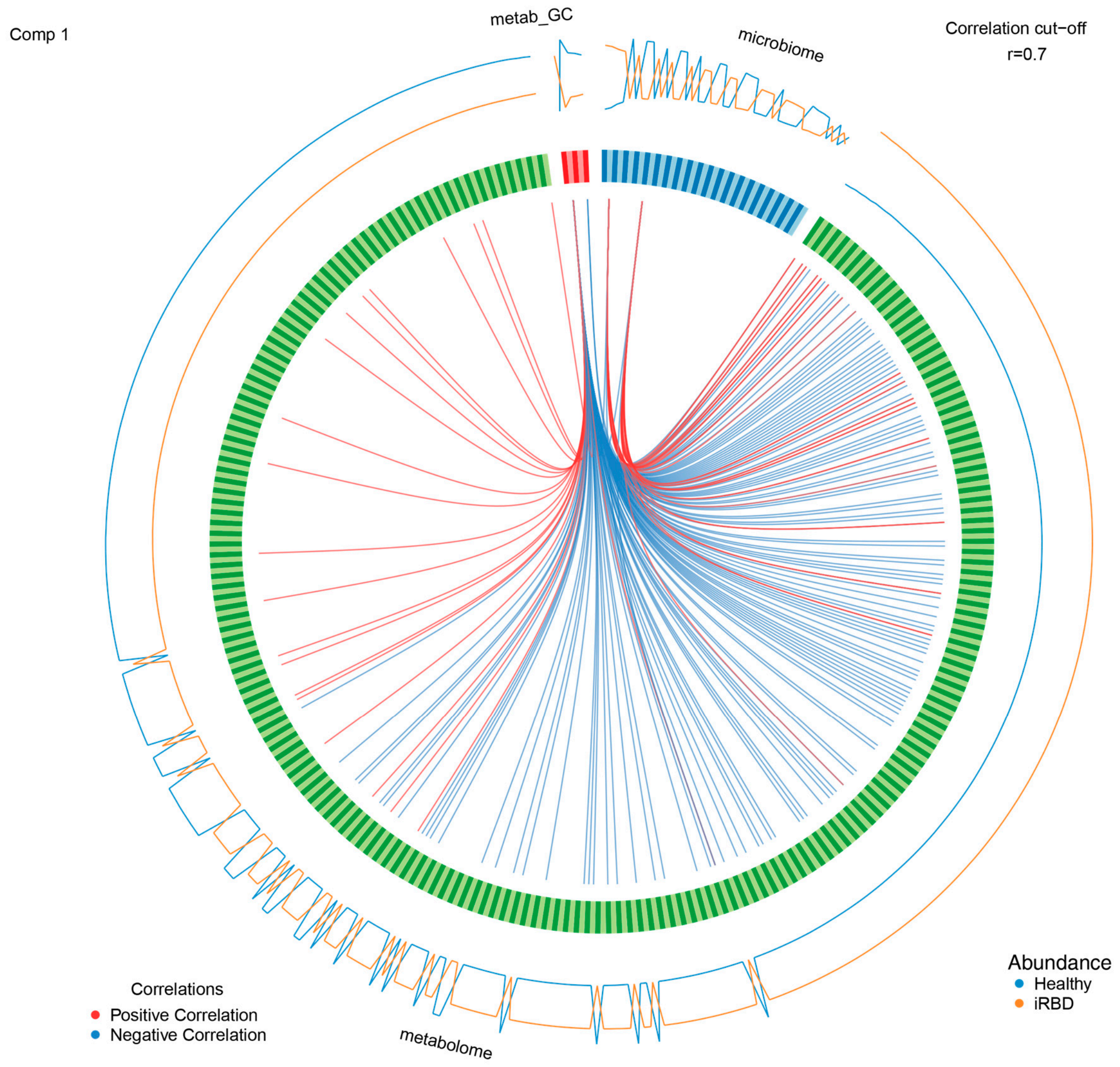

Figure S6. Circos plot of the multiblock sparse Partial Least Squares Discriminant Analysis (sPLS-DA) generated using the mixOmics package. Only associations with an absolute correlation coefficient $|r| \geq 0.7$ are shown. The outer circular tracks display the mean normalized abundance of each variable in the two study groups (blue, HC; orange, iRBD). Inner links represent pairwise correlations between variables across the three integrated datasets, with red and blue lines indicating positive and negative correlations, respectively. $n = 33$ (19 iRBD, 14 HC).
